# COVI-Prim Longitudinal Survey: Experiences of Primary Care Physicians During the Early Phase of the COVID-19 Pandemic

**DOI:** 10.3389/fmed.2022.761283

**Published:** 2022-02-21

**Authors:** Dagmar Schaffler-Schaden, Karola Mergenthal, Alexander Avian, Sebastian Huter, Ulrike Spary-Kainz, Herbert Bachler, Maria Flamm, Andrea Siebenhofer

**Affiliations:** ^1^Institute for General Practice, Family Medicine and Preventive Medicine, Paracelsus Medical University, Salzburg, Austria; ^2^Institute of General Practice, Johann Wolfgang Goethe University Frankfurt, Frankfurt, Germany; ^3^Institute for Medical Informatics, Statistics and Documentation, Medical University Graz, Graz, Austria; ^4^Institute of General Practice and Evidence Based Health Services Research, Medical University Graz, Graz, Austria; ^5^Institute of General Practice, Medical University Innsbruck, Innsbruck, Austria

**Keywords:** general practitioner, COVID-19 pandemic, primary care, longitudinal survey, work load, self-confidence, worries, interactions with patients and colleagues

## Abstract

**Background:**

General practitioners (GPs) are the mainstay of primary care and play a critical role in pandemics. During the first wave of the COVID-19 pandemic, this international study aimed to explore changes in the workload of general practitioners, as well as their interactions with patients and colleagues, and their self-confidence and concerns.

**Methods:**

An online survey was conducted among GPs in Austria and Germany. Participants were asked to answer a basic questionnaire and participate in a subsequent longitudinal survey containing closed and open-ended items. All data were pseudonymized.

**Results:**

Overall, 723 general practitioners from Austria and Germany took part in the longitudinal survey over a period of 12 weeks (April 3–July 2, 2020). The majority of GPs had less direct contact with patients at the beginning of the survey (96 vs. 49% at the end of the study period). At first, doctors were mainly concerned with pandemic-related issues and had to care for the patients of GP colleagues that were in quarantine, which meant they had less time for routine work such as screenings and treating chronic diseases. Over the survey period, GPs' self-confidence increased and their concerns about income loss decreased.

**Conclusions:**

Following a difficult initial phase when protective equipment and information were lacking, physicians in primary care adapted quickly to new situations. Experience with telemedicine should help them face future challenges and may help prevent a decline in the delivery of routine health care and care for chronically ill patients.

**Registration:**

Trial registration at the German Clinical Trials Register: DRKS00021231.

## Introduction

The COVID-19 pandemic has created a special situation for general practitioners (GPs) throughout the world. Although challenges undoubtedly vary between countries, the majority of GPs have probably experienced an unusual and unexpected burden. The role of GPs as gatekeepers in the front line of primary care is crucial in a pandemic, as, by maintaining care for those with acute or chronic conditions, they help ensure that hospital and intensive care beds are available for the critically ill (1). To enable treatment without face-to-face contact, many GPs had to reorganize working processes in their practices and install new technologies (2, 3). In many countries telehealth was implemented as a result of the pandemic. Beginning with the first wave, GPs in Austria dispensed electronic prescriptions, which they transmitted directly to the pharmacies their patients frequented. During this initial phase, it also became possible for patients to demand sick leave certificates by telephone without the need to first be examined. Hopefully, such facilitated services for patients and doctors will remain in place when the pandemic is over (4).

The results of the COVI-Prim-Start cross-sectional survey revealed that primary care physicians were unprepared in the first few weeks of the pandemic. Despite the importance of their work, they lacked personal protective equipment (PPE) and information on where to obtain materials (5, 6). Unlike hospitals, GPs often have no contingency plans for emergency situations. In previous pandemics, governments were therefore called upon to support primary care physicians by purchasing materials for them (7). The COVI-Prim-Start survey showed that a reticence among patients to come to the practice changed the structure of GPs' work and led to a substantial increase in telephone and email-contacts (8). The survey also showed that 60% of GPs were concerned that a decrease in services provided to patients would lead to lost income and jeopardize the future of their practices and employees (5). Furthermore, GPs had higher levels of anxiety than hospital staff, which was attributable to higher perceptions of risk resulting from a shortage of PPE (9).

The primary aim of this international longitudinal study was to examine GPs' workload, their interactions with patients and colleagues, as well as their self-confidence and concerns, during a 12-week period in the first wave of the pandemic. We further investigated the impact of sex, age, country, size of the town in which the practice was located, and ownership status.

## Materials and Methods

This manuscript was prepared in accordance with the CHERRIES criteria (10). COVI-Prim-Long is part of the international COVI-Prim project (5) whereby participants were invited to answer a basic questionnaire, followed by further questionnaires that were then sent to participating GPs at regular intervals. The methods and design of the underlying COVI-Prim study are described in detail elsewhere (5).

For the longitudinal study, we selected from the initial baseline COVI-Prim item pool those items for which responses were expected to change over the course of the pandemic. A group of experts (AA, AS, DSS, HB, KM, MF, SH) consisting of GPs and a psychometrician selected items that covered three general topics: (1) GPs' workload, (2) GPs' interactions with patients and colleagues and (3) GPs' self-confidence and worries. The final longitudinal questionnaire consisted of 15 items, of which three required GPs to provide exact numbers, seven required them to provide percentages, and five were open-ended (3 pages) (5).

### Survey

The questionnaire was transferred to Lime Survey®. Following completion of the German version of the COVI-Prim baseline survey, respondents were invited to take part in the longitudinal study. Respondents that were willing to participate first had to provide an individual pseudonymization code (first two letter of mother's first name + first two letters of father's first name + day of mother's date of birth + year of father's date of birth). Afterwards, they provided their e-mail addresses. The e-mail addresses and the survey responses were stored separately, so that they could not be linked. The longitudinal study consisted of eight follow-up surveys. In April and at the beginning of May 2020, the follow-up surveys were conducted on a weekly basis. Afterwards, follow-up surveys were carried out at two-weekly intervals (follow-up survey period 1: 10.4–16.4.2020; response rate: 39%; follow-up survey period 2: 17.4–23.4.2020; response rate: 55%, follow-up survey period 3: 24.4–30.4.2020; response rate: 45%; follow-up survey period 4: 1.5–7.5.2020; response rate: 44%; follow-up survey period 5: 8.5–21.5.2020; response rate: 39%; follow-up survey period 6: 22.5–4.6.2020; response rate: 38%; follow-up survey period 7: 5.6–18.6.2020; response rate: 39%; follow-up survey period 8: 19.6–2.7.2020; response rate: 30%). Participation was voluntary and no incentives were offered. After the survey was finished, all data on the online platform were stored in SPSS files.

### Statistics

Baseline characteristics are presented as median (min-max) or mean ± SD (standard deviation), as appropriate. Categorical variables are given as absolute numbers and in percent. The baseline characteristics of GPs that only answered the baseline survey and those that also answered follow-up surveys were compared using the chi-square test, *t*-Test or Mann Whitney *U*-test as appropriate.

In the main analysis, changes in the responses to each item over time were analyzed using generalized mixed models for binary outcomes (PROC GLIMMIX). Responses were categorized as agreement (“yes” and “probably”) and disagreement (“probably not” and “no”), as well as high burden (“high” and “very high”) and low burden (“very low,” “low,” and “moderate”). A first-order autoregressive covariance structure was used in all models. The autoregressive covariance structure assumes systematically decreasing correlation with increasing distance between time points. Adjacent time points will therefore have the highest correlations. In a second step, potential influencing factors [age, sex, country of survey (Germany vs. Austria), size of town of practice (<5,000 vs. 5,000—<20,000 vs. 20,000—<1,00,000 vs. ≥1,00,000), type of practice (single-handed vs. group practice) position in the practice (employee vs. owner)] were included in the models separately, and further details presented if the differences were statistically significant. *P*-values for the fixed effects of time were indicated with the subscript “week” (*p*_week_), and for the fixed effect of the included variable (e.g., sex) with the name of the variable (e.g., *p*_sex_). Interactions between these two effects were indicated with the subscript week and the name of the variable (e.g., $p_{\text{se}{\text{x}}^{*}week}$). Bonferroni correction was used to adjust for multiple testing. Results were presented using estimated means and 95% confidence intervals (95%CI). No statistical correction was carried out to account for non-representative samples. A *p*-value < 0.05 was considered statistically significant. Statistical analyses were performed using SAS 9.4 (SAS Institute Inc., Cary, NC, USA).

### Ethics

The study protocol was approved by the local ethics committee of Goethe University Frankfurt, Germany (20-619).

The study was conducted by the Medical University in Graz, the Paracelsus Medical University in Salzburg and Goethe University in Frankfurt. This research received no specific grant from any funding agency in the public, commercial or non-profit sectors.

## Results

**Figure 1:**
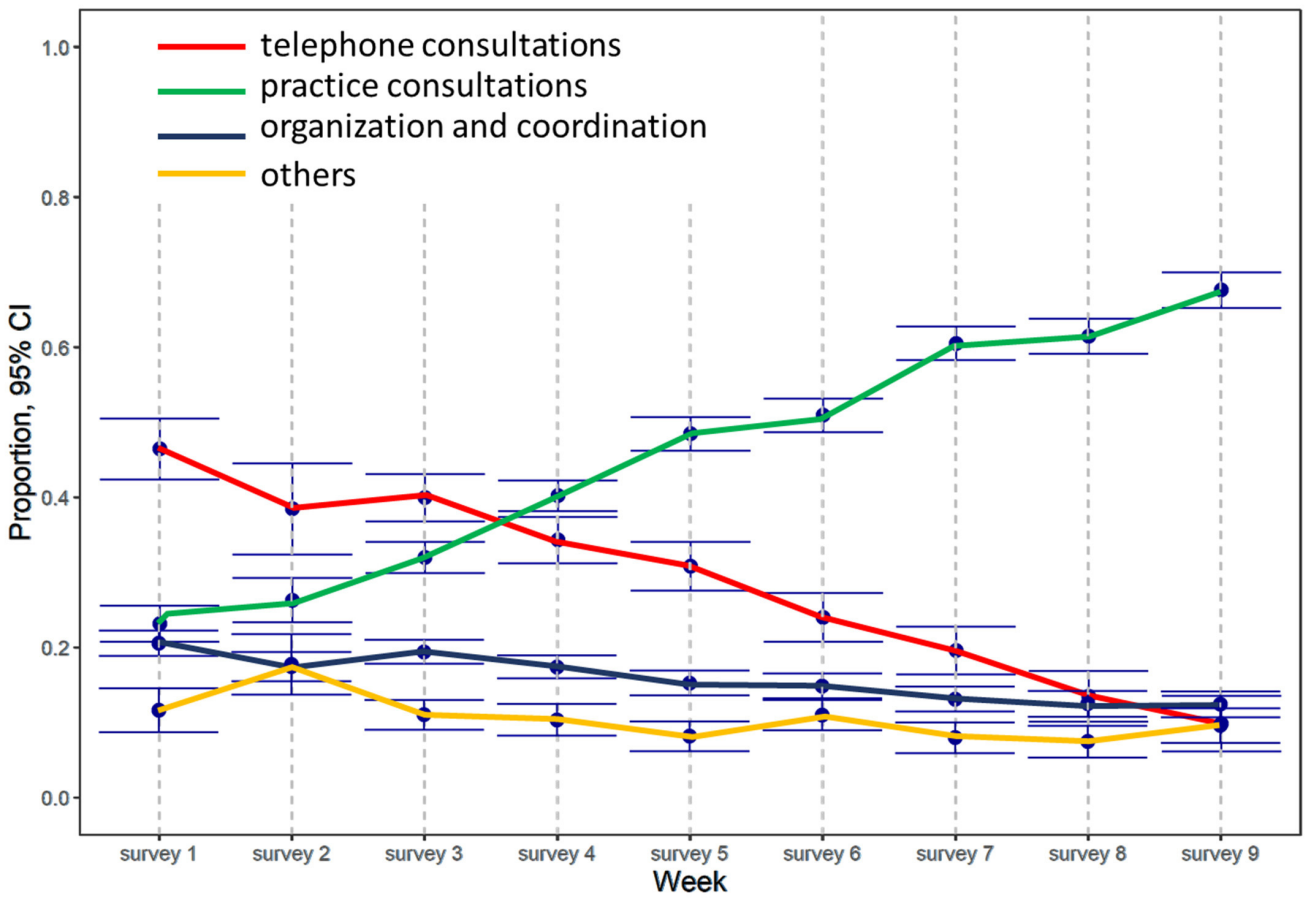
Presentation of received answers.

Overall, 2,836 (5,877 responses) Austrian and German GPs answered the baseline survey during the first phase of the COVID-19 pandemic (April 3–May 29) (see Figure 1). Of these, 723 (2,815 responses) also responded to at least one follow-up survey. The median number of answered surveys was 3 (Interquartile range: 1–4).

Mean age of the GPs was 52.2 years (SD: 9.0), the majority were male, practiced in a city with fewer than 20,000 inhabitants, had a single-handed practice, were owners of the practice and came from Germany. All demographic characteristics are provided in Table 1. A comparison of GPs only answering the baseline survey and those answering at least one follow-up survey revealed no significant differences in terms of sex, age, the size of town in which the practice was based, and the position of the respondent in the practice. GPs working in a single-handed practice (23.9 vs. 29.2%) and GPs from Austria (23.7 vs. 27.9%) were less likely to answer more than one survey.

**Table 1:** Baseline demographics.

	**All**
	***n* = 723**
Age (years)	52.2 ± 9.0
Sex	
Male	332 (45.9%)
Female	314 (43.4%)
Other	2 (0.3%)
Missing	75 (10.4%)
Size of town of practice	
<5,000	181 (25.0%)
5,000–<20,000	212 (29.3%)
20,000–<1,00,000	108 (14.9%)
≥100,000	147 (20.3%)
Missing	75 (10.4%)
Type of practice	
Single-handed	344 (47.6%)
Not single-handed	304 (42.0%)
Missing	75 (10.4%)
Position in the practice	
Employee	58 (8.0%)
Owner	584 (80.8%)
Locum	6 (0.8%)
Missing	75 (10.4%)
Year practice was set up	Median: 2005 Range: 1975–2020
Country	
Austria	242 (33.5%)
Germany	406 (56.2%)
Missing	75 (10.4%)

### GPs' Workload

**Figure 2:**
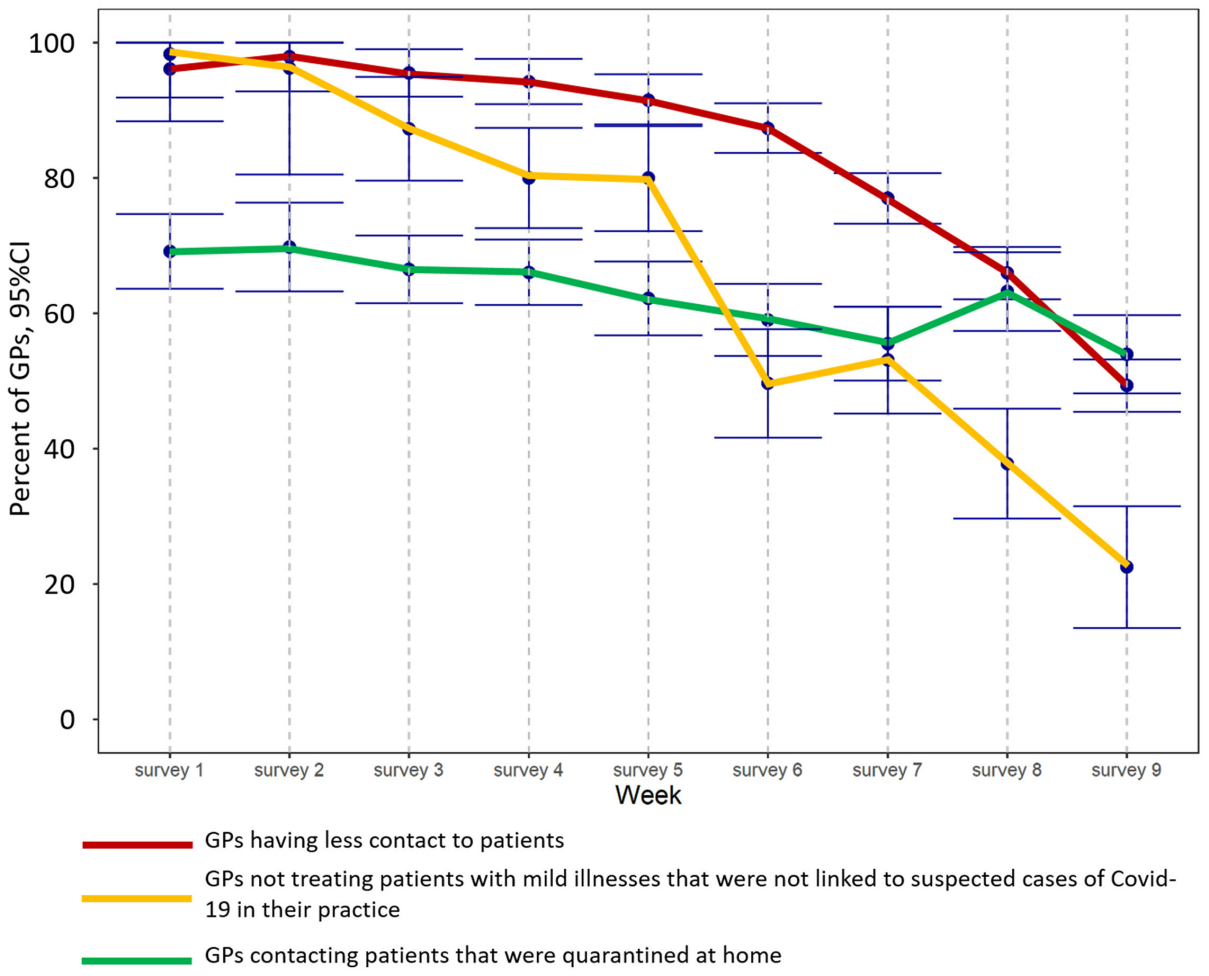
Change in type of work performed during the study period.

Although the number of hours worked per week remained unchanged during the course of the study (*p* = 1.000), the content of the work changed over time (see Figure 2). At the beginning of our survey, almost half the working hours (46%, 95%CI: 41–51%) were directly or indirectly linked to the pandemic and about one third (37%, 95%CI: 33–40%) were spent in routine care such as screening or treating chronically ill patients. At first, the share of telephone consultations was high (46%, 95%CI: 42–51%), and the proportion of practice consultations low (23%, 95%CI: 0.21–26%). However, the situation changed during the observation period (see Figure 2), and when the final measurement occurred, the amount of time spent on telephone consultations had dropped to 10% (10%, 95%CI: 6–14%; *p*_week_ < 0.001), and that spent in practice consultations had increased to two-thirds (68, 95%CI: 65–70%; *p*_week_ < 0.001). In the final survey, only 10% of working hours (11%, 95%CI: 7–16%; *p*_week_ < 0.001) were directly or indirectly linked to the pandemic, while about three-quarters (72, 95%CI: 68%−0.75; *p*_week_ < 0.001) were spent on routine care such as screening or treating chronically ill patients. The time spent on organization and coordination decreased from 21% (95%CI: 19–22%) to 12% (95%CI: 1114%; *p*_week_ < 0.001) at the end of the survey. The number of GPs rating the overall burden of their work as high or very high varied between 23 and 36% before the end of May, but afterwards increased to 56% (95%CI: 51–61%; *p*_week_ < 0.001).

#### Sex Differences

Male GPs worked more hours per week than female GPs (*p* < 0.001), spent less time on telephone consultations (*p* = 0.042), less time providing care that was directly or indirectly linked to the pandemic (*p* = 0.006) and correspondingly more on routine care such as screening or treating chronically ill patients.

#### Age Differences

While at the beginning of the pandemic the proportion of practice consultations rose in line with the age of GPs, the influence of age had decreased by the end of the survey ($p_{\text{ag}{\text{e}}^{*}week}$ <0.001). The same is true for the proportion of working hours spent on organization and coordination, which increased with age at the beginning of the pandemic, but was no longer influenced by age at the end of the survey ($p_{\text{ag}{\text{e}}^{*}week}$ < 0.001).

#### Differences Between Austria and Germany

While German GPs worked more than Austrian GPs in April 2020, the number of hours worked in the two groups was comparable in May and June ($p_{\text{countr}{\text{y}}^{*}week}$ <0.001). Overall, Austrian GPs spent more working time on telephone consultations (*p*_country_ <0.001) and less on practice consultations (*p*_country_ <0.001). This difference was more pronounced at the beginning of the pandemic (telephone: beginning: A: 54%, 95%CI 51–57% vs. G: 36%, 95%CI 32–39%; end: A: 15%, 95%CI 4–25% vs. G: 8%, 95%CI 0–18%; $p_{\text{countr}{\text{y}}^{*}week}$: *p* < 0.001; practice: beginning: A: 15%, 95%CI 725% vs. G: 28%, 95%CI 19–37%; end: A: 66%, 95%CI 51–80% vs. G: 67%, 95%CI 53–81%; $p_{\text{countr}{\text{y}}^{*}week}$: *p* = 0.008). The proportion of working time spent on organization and coordination was higher in Germany throughout the whole study period (*p*_country_ <0.001). Overall, the proportion of working time linked directly or indirectly to COVID-19 was higher in Germany (*p*_country_ = 0.004), and the time spent on routine care correspondingly lower (*p*_country_ < 0.001).

#### Differences Associated With the Size of Town

Overall, the proportion of working time spent on organization and coordination was highest among GPs whose practices were located in towns with at least 1,00,000 inhabitants, followed by GPs in towns with fewer than 5,000 inhabitants and towns with 5,000–19,000 inhabitants (*p*_citysize_ = 0.044). GPs in towns with at least 1,00,000 inhabitants spent the most working time on tasks linked directly or indirectly to COVID-19. For the three other groups the proportion was comparable (*p*_citysize_ = 0.028).

#### Differences According to Ownership Status

GPs working in their own practices worked more than GPs working as employees over the whole study period (*p*_position_ <0.001). They also spent a higher proportion of their working time on organization and coordination (*p*_position_ = 0.012).

### GPs' Interactions With Patients and Colleagues

Overall, the number of GPs whose contact to patients fell as a result of the pandemic decreased from 96% (95%CI: 92–100%) to 49% (95%CI: 45–53%; *p*_week_ <0.001) by the end of the observation period. The proportion of GPs that contacted patients in home quarantine in order to monitor the progression of the disease decreased from 69% (95%CI: 64–75%) at the beginning to 54% (95%CI: 48–60%; *p*_week_ <0.001) at the end of the survey. The proportion of GPs that did not treat patients with mild illnesses that were not linked to suspected cases of COVID-19 in their practice, and that provided such care by phone or online, decreased from almost 100% (98%, 95%CI: 89–100%) to 23% (95%CI: 14–31%; *p*_week_ <0.001) over the observation period.

**Figure 3:**
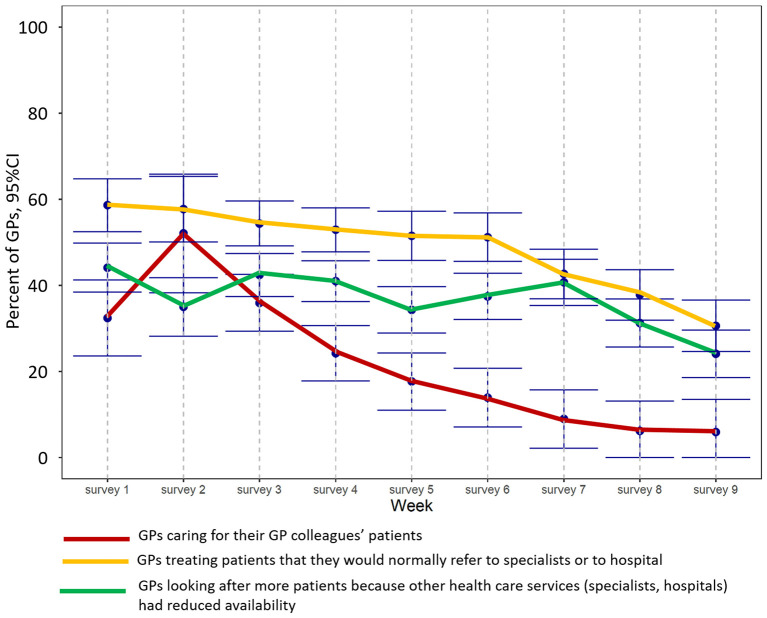
Interaction of GPs with patients during the study period.

**Figure 4:**
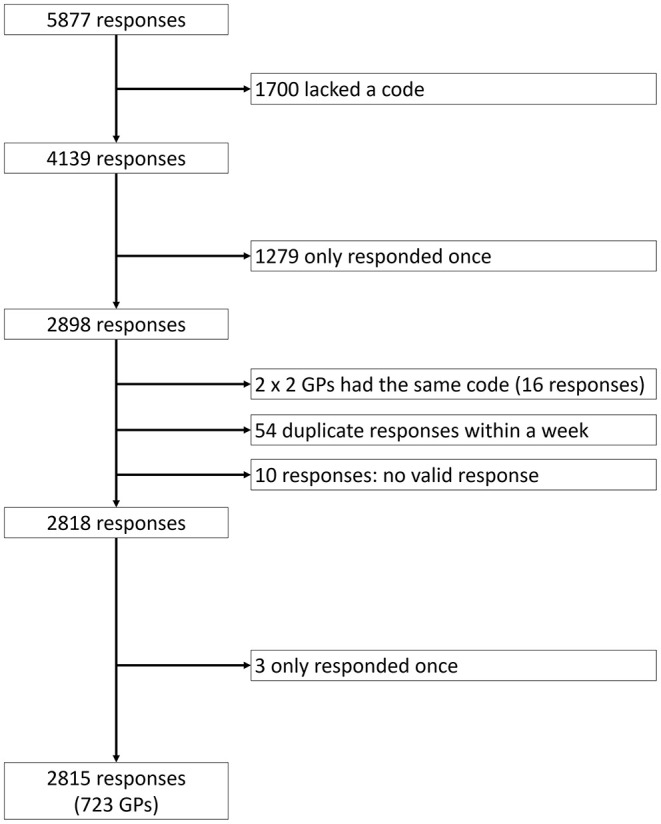
Interaction of GPs with their GP colleagues during the study period.

In the first few weeks of the survey, the number of GPs caring for the patients of GP colleagues that had closed their practices because they were in quarantine increased from 32% (95%CI: 24–41%) to 52% in week 2 (95%CI: 38–66%), but had decreased to 6% (95%CI: 0–13%; *p*_week_ <0.001) by the final survey (see Figure 4). The number of GPs that had to look after more patients than usual because other health care services (specialists, hospitals) had reduced their availability ranged from 34 to 44% at the end of May but decreased to 24% (95%CI: 19–30%; *p*_week_ < 0.001) in June. Furthermore, the number of GPs treating patients they would normally refer to specialists or to hospital decreased from 59% (95%CI: 52–64%) to 31% (95%CI: 25–37%; *p*_week_ < 0.001) over the period (Figure 3).

#### Differences Between Austria and Germany

Throughout the study period, the proportion of GPs that contacted patients in home quarantine in order to monitor the progression of the disease was higher in Germany (*p*_country_ =0.002) than in Austria. In Germany, the number of GPs that had to take on patients from GP colleagues that had been required to close their practices because they were in quarantine was lower than in Austria (*p*_country_ <0.001), as was the number of GPs treating patients they would normally have referred to specialists or to hospital (*p*_country_ =0.006).

### GPs' Self-Confidence and Worries

The proportion of GPs that felt helpless when caring for patients with COVID-19 and that were sometimes unsure whether they were doing everything right when treating such patients decreased during the course of the longitudinal survey (helpless: from 25%, 95%CI: 20–29% to 8%, 95%CI: 4–12%, *p*_week_ <0.001; unsure: from 55%, 95%CI: 44–66% to 22%, 95%CI: 11–32%, *p*_week_ <0.001). Furthermore, the proportion of GPs that were worried they might unknowingly infect their patients and that were worried about how the pandemic would affect their and their employees' economic outlook decreased during the course of the longitudinal survey (infect patients: from 56%, 95%CI: 45–66% to 22%, 95%CI: 12–31%, *p*_week_ =0.008; economic outlook: from 58%, 95%CI: 52–64% to 32% 95%CI: 26–37%, *p*_week_=0.006).

#### Sex Differences

Overall, the proportion of male GPs that were unsure whether they were doing everything right when treating patients that had been infected with Covid-19 was lower than among female GPs (p_sex_ =0.020). At the beginning of the survey, the proportion of male GPs that worried about how the pandemic would affect their and their employees' economic outlook was lower than among female GPs (male: 51%, 95%CI: 43–59%; female: 65%, 95%CI: 57–73%), and decreased slightly over time. However, the decrease in the proportion of female GPs was steeper, and the proportion in the final survey was actually lower among female than among male GPs (male: 35%, 95%CI: 28–43%; female: 28%, 95%CI: 20–36%; $p_{\text{se}{\text{x}}^{*}week}$ =0.040).

#### Differences Between Austria and Germany

Irrespective of time-point, the proportion of GPs in Germany that worried about how the pandemic would affect their and their employees' economic outlook (*p*_country_ <0.001) and that worried that they might unknowingly infect their patients (*p*_country_ <0.001) were higher than in Austria.

## Discussion

Our study shows how the workload of GPs, their interactions with patients and colleagues, their self-confidence and their concerns changed in Austria and Germany over a period of 12 weeks during the first phase of the COVID-19 pandemic. The study was launched in April 2020, when the first wave of the pandemic was in full swing and both countries were in the midst of their first lockdowns^1^. The high level of responses—each GP participated in approximately three surveys—encouraged us to ask GPs to answer the same questions on eight separate occasions.

Even though the burden of work increased, overall weekly working times remained unchanged over the whole observation period. Although GPs spent almost half their working time on pandemic-related issues at the beginning of the survey, their work situation had normalized by June 2020 and the number of screenings, and examinations of chronically ill patients, had recovered. With regard to GPs' interactions with patients, it would appear that although contacts to patients fell to almost zero at the beginning of the survey, overall workload remained unchanged, with the time spent on telephone consultations particularly high (Figure 2). Participating GPs also provided care to patients of their GP colleagues that had closed their practices because they were in quarantine, and they had to treat patients that might normally have been admitted to hospital or referred to a specialist (Figure 3). Overall, GPs' self-confidence strengthened and their worries decreased during the observation period.

Regarding country-specific differences, German GPs were more involved in monitoring patients in home quarantine throughout the study period. This may be because COVID-teams were available in Austria to visit infected patients at home and refer them to hospital if necessary. This public measure was taken in order to prevent GPs from having to close their practices due to infections and thus from being unable to provide primary care. In Austria, suspected cases were initially referred to testing centers, while in Germany, GPs started testing patients in their practices earlier. This may explain why, at the beginning of the pandemic, German GPs had a higher workload and Austrian GPs provided more telephone consultations. Practice owners had a higher workload throughout the entire study period, which is not surprising since the owner is usually responsible for the smooth running of the practice, while employees have limited working hours. However, GPs adapted quickly and efficiently to the new situation (5). GPs' work content also changed during the study period, with more than half the GPs rating their workload as high or very high by the end.

Although the number of face-to-face contacts with patients declined as a result of the new situation, the actual workload did not. Routine tasks such as screening and treating chronic diseases played a minor role in the first phase of our survey. As the pandemic progressed, face-to-face contact with patients and the reasons for consultations returned to normal over the study period, which may reflect the steady decrease in the incidence of COVID-19 (see text footnote 1). At the beginning of the pandemic, only limited options were available for patients with chronic diseases to meet their GPs. However, GPs worked hard to ensure the earliest possible resumption of care for their chronically ill patients.

Communication between doctors and patients is essential and can be effective in reducing patient anxiety and improving health outcomes (11). Several authors have addressed the problem of missed or postponed primary care for acute diseases and cancer during the pandemic (12, 13), and it is assumed that the effects of neglected diagnostic investigations and therapy will become more visible in the near future (8).

In the first wave of the pandemic, many patients refrained from taking part in face-to-face consultations either because they were encouraged to stay at home, they did not want to “bother” their doctor at such a difficult time, or for fear of infection (14). This resulted in a sudden switch to telemedical consulting among GPs in Austria and Germany, as well as in other countries (15). In Austria and Germany, health insurance companies set new tariffs for the reimbursement of telemedicine consultations to support this trend. Before the pandemic, many efforts to strengthen telemedicine in primary care failed for ethical or legal reasons. Although the pandemic led to the introduction of cost reimbursement for telehealth in several countries, countries with a poor infrastructure were not in a position to take advantage of these opportunities (16). The number of patient contacts via phone, email and video was high at the beginning of the pandemic (Figure 2), but despite the advantages offered by such technical options, most patients still prefer face-to-face contact with their doctors, as indicated by the decline in telephone- and video consultations over the study period (Figure 2). This may partly reflect such barriers to telemedicine as a lack of experience in using remote technologies, a lack of resources, or older age (17). Structured guidelines for a remote assessment of symptoms can help doctors identify red flags early (3).

As entrenched reimbursement models did not consider remote medical services, primary care physicians in many countries were concerned that the COVID-19 pandemic might lead to a drop in their incomes (18). In Austria, telephone and video consultations are now reimbursed in the same way as a personal consultation in a GP's practice (19). In Germany, reimbursement for telephone and video consultations has been increased but remains lower than for consultations in the practice (20). However, one requirement for remuneration is that the patient is known to the GP. On the other hand, strict data protection regulations, restricted budgets, and a reluctance to invest in the new equipment required to provide distance consultations, may have reduced the willingness of GPs to implement video consultations, especially in the early days. Public support measures such as short-time work and tax relief were realized earlier in Austria than in Germany. Stakeholders also reacted to economic concerns by providing financial support for certain medical services in advance.

As the incidence of infections dropped, patients returned to the practice and concerns about economic problems diminished among survey participants. In the beginning, German GPs and specialists were more worried about empty waiting rooms than their Austrian counterparts because of differences in the countries' health insurance systems. In Austria, all patients have statutory health insurance, while in Germany, a substantial portion of patients are privately insured. The absence of self-paying and privately insured patients during the first wave of the pandemic may be one reason why German GPs were more concerned about their economic outlook than Austrian GPs. Continuous improvements in knowledge and organization probably helped raise GPs' confidence in their ability to deal with the disease. It is well known that GPs and other health care workers caring for patients during epidemics and pandemics are at risk of experiencing fear, anxiety, stress and depression (21, 22). In addition, a fear of becoming ill themselves may reduce their willingness to treat infected patients (23). Greater uncertainty among female general practitioners in the treatment of COVID patients in our study is consistent with other study results and confirms that female physicians are generally significantly less self-confident with respect to their competencies (24). In our study, the majority of practice owners were male, which is probably because female doctors are more often employed and work part time due to family obligations (25).

Increasing confidence in infection control may have protective effects in terms of reducing worries and stress (26). Our findings confirm this effect and suggest that most practices and the general population became accustomed to dealing with the pandemic over the study period. Growing knowledge about transmission routes, effective testing strategies and protective measures against infection, led to a significant decrease in fears of contracting the disease. Centralized crisis management and organization of PPE should be considered in future pandemics. Since many GPs work in a single-handed practice, early public support could relieve them of some of the pressures of dealing with such a situation. Encouraging the general population and especially chronically ill patients to keep their scheduled appointments for health check-ups despite a pandemic would also help avoid collateral damage.

### Strengths and Limitations

Our study has some limitations. Firstly, in order to distribute the questionnaire when the situation was at its most acute, it was developed in a very short time. Despite our best efforts, the questionnaire may therefore not have covered all relevant aspects. Secondly, we could not calculate the response rate because a systematic area-wide survey was not possible in the time frame we allotted ourselves. However, the number of responses to the longitudinal survey, which involved the same questions being asked repeatedly over a 2-month period, far exceeded our expectations, especially considering the difficulties that are usually encountered in recruiting GPs for research projects (27). On average, each GP answered around three of the eight surveys, and there was no significant difference between the baseline characteristics of GPs answering only the baseline questionnaire and those responding to at least one follow-up survey (Age: *p* =0.580, sex: *p* =0.083). Furthermore, differences were found that indicate that GPs lost to FU are less affected by the pandemic (Supplementary Table 1). Since we have found some differences between GPs lost to follow up and GPs that have responded to follow up surveys, we cannot rule out that an attrition bias has occurred. According to Sedgwick (28) an attrition bias occurs, “when people are lost to follow-up in a non-random manner.” If lost to follow is not random, there is a risk that those who continue to participate differ from the GPs lost to follow up in certain characteristics that may also have an impact on the outcome. Thirdly, since the recruitment process exploited regional networks and professional associations, the selection of participants may not have been representative for Austria and Germany as a whole and therefore a selection bias may have occurred. A selection bias arises when there are differences in the probability that particular persons will take part in the survey. A common reason for selection bias in online surveys is the need to have access to the internet. While this is unlikely to be a factor for GPs, differences in motivation to participate and the fact that not all GPs in Austria and Germany were contacted may have led to a selection bias. In the literature, education and socioeconomic status are repeatedly associated with selection bias (29, 30). However, as the group of persons investigated in this study is similar in terms of education and socioeconomic status, it is unlikely to have played a role. Among GPs, Verger et al. (31) showed that GPs responding to the first invitation to participate in an online survey differ significantly from GPs that answer an online survey only after several reminders, after receiving a reminder by telephone, or who respond to the survey during an interview when no reminder has been successful. Differences could be observed in gender, age, workload, a readiness to occasionally practice complementary and alternative medicine, confidence that the Ministry of Health will ensure that vaccines are safe and correctly assess the danger presented by COVID-19. Furthermore, as with any study, it must also be taken into account that an interpretation bias could occur. Of the different types of interpretation bias, the confirmation bias is the most likely to occur in this study. Since part of the study team works in the field of general medicine and is therefore in daily contact with general practitioners, certain topics may have been given too much space in the discussion. Other possibly equally important topics could therefore not have been adequately discussed. A further limitation is that our survey was only carried out among GPs and did not involve other practice team members from a primary care setting.

## Conclusion

Due to the immense challenges posed by the pandemic, GPs had to adapt quickly and competently to changing situations. GPs offered more telephone consultations in the uncertain initial phase of the pandemic, but increasingly returned to face-to-face contacts afterwards. During the first wave of the pandemic, initial worries about handling the infection disappeared, self-confidence increased, and the use of new technical methods quickly became established. Lessons learned from the current pandemic can help increase GPs' ability to deal with possible similar events in the future. The COVID-19 pandemic has probably helped improve care by overcoming reservations about the use of telemedicine.

## Data Availability Statement

The raw data supporting the conclusions of this article will be made available by the authors, without undue reservation.

## Ethics Statement

The studies involving human participants were reviewed and approved by Ethics Committee of Goethe University Frankfurt, Germany. Written informed consent for participation was not required for this study in accordance with the national legislation and the institutional requirements.

## Author Contributions

DS-S, KM, AA, SH, US-K, HB, MF, and AS: conceptualization, investigation, methodology, writing original draft, and writing review and editing. SH and AA: data curation. AA: formal analysis and visualization. DS-S, KM, SH, MF, and AS: project administration. AS and KM: resources. AS and MF: supervision. All authors contributed to the article and approved the submitted version.

## Conflict of Interest

The authors declare that the research was conducted in the absence of any commercial or financial relationships that could be construed as a potential conflict of interest.

## Publisher's Note

All claims expressed in this article are solely those of the authors and do not necessarily represent those of their affiliated organizations, or those of the publisher, the editors and the reviewers. Any product that may be evaluated in this article, or claim that may be made by its manufacturer, is not guaranteed or endorsed by the publisher.

## Footnotes

^1^https://ourworldindata.org/explorers/coronavirus-data-explorer?zoomToSelection=true&time=2020-03-05..2020-06-04&pickerSort=desc&pickerMetric=new_cases_per_million&Metric=Confirmed+cases&Interval=7-day+rolling+average&Relative+to+Population=true&Align+outbreaks=false&country=AUT~DEU.
