# Supplementary material for: COVI-Prim Longitudinal Survey: Experiences of Primary Care Physicians During the Early Phase of the COVID-19 Pandemic
Source: Front Med (Lausanne). 2022 Feb 21;9:761283. doi: 10.3389/fmed.2022.761283 (PMC8898947; doi:10.3389/fmed.2022.761283)
Supplement: Supplementary file 1 [file Data_Sheet_1.docx]

Supplementary Material

# Supplementary Figures*.*

Supplemental Figure 1.

Courses of GP’s work load over time. Significant differences in the main effects sex, country, position and size of town and significant different courses depending on these variables (interaction) are shown. It is indicated below each figure whether the main effect or the interaction is significant. If no significance was observed, no figure is shown.

| Sex  week*sex | | Country  week * country | | position  week*position | | size of town  week*size of town |
| --- | --- | --- | --- | --- | --- | --- |
| How many hours did you work last week? | | | | | | |
| 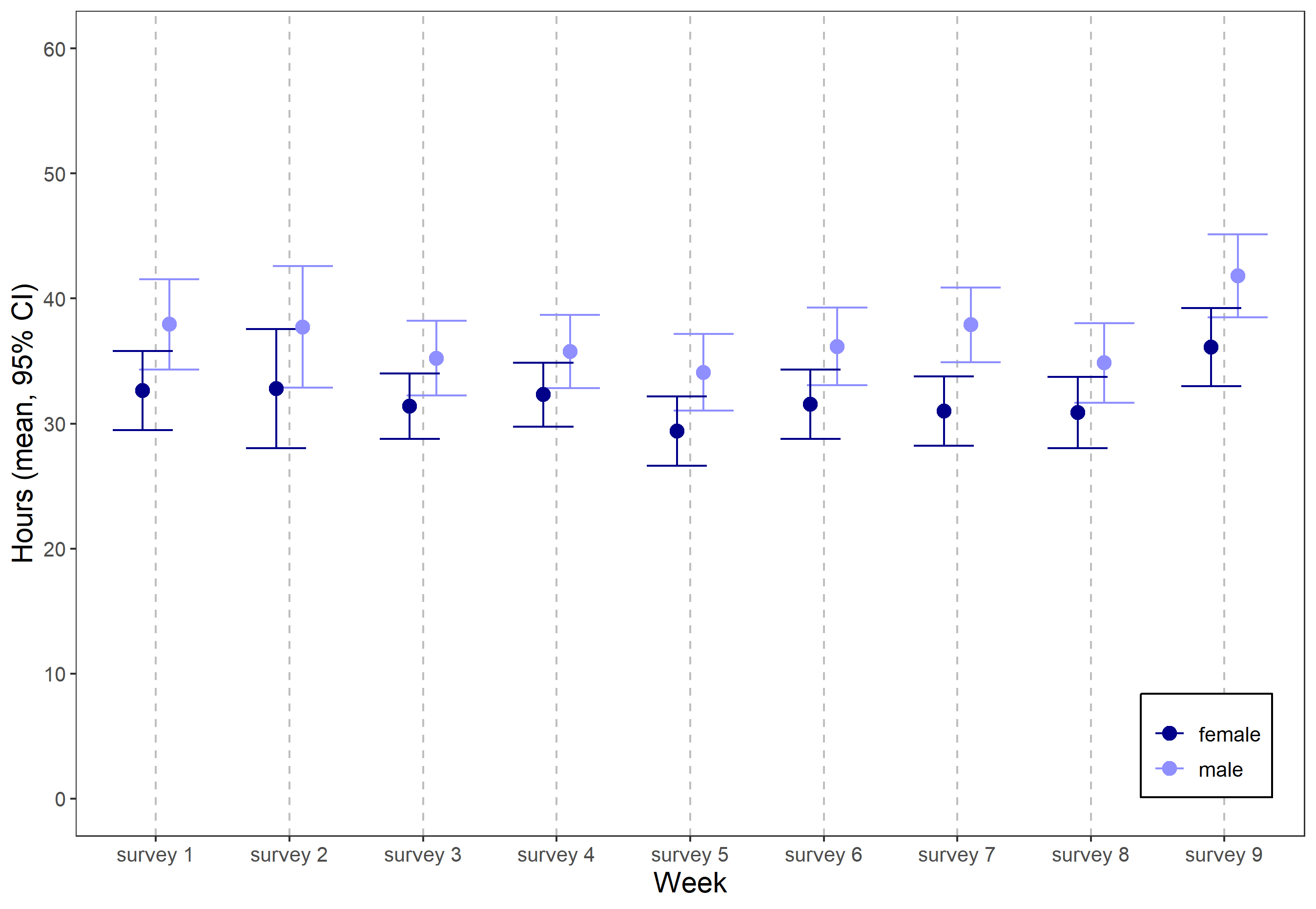  Sex | | 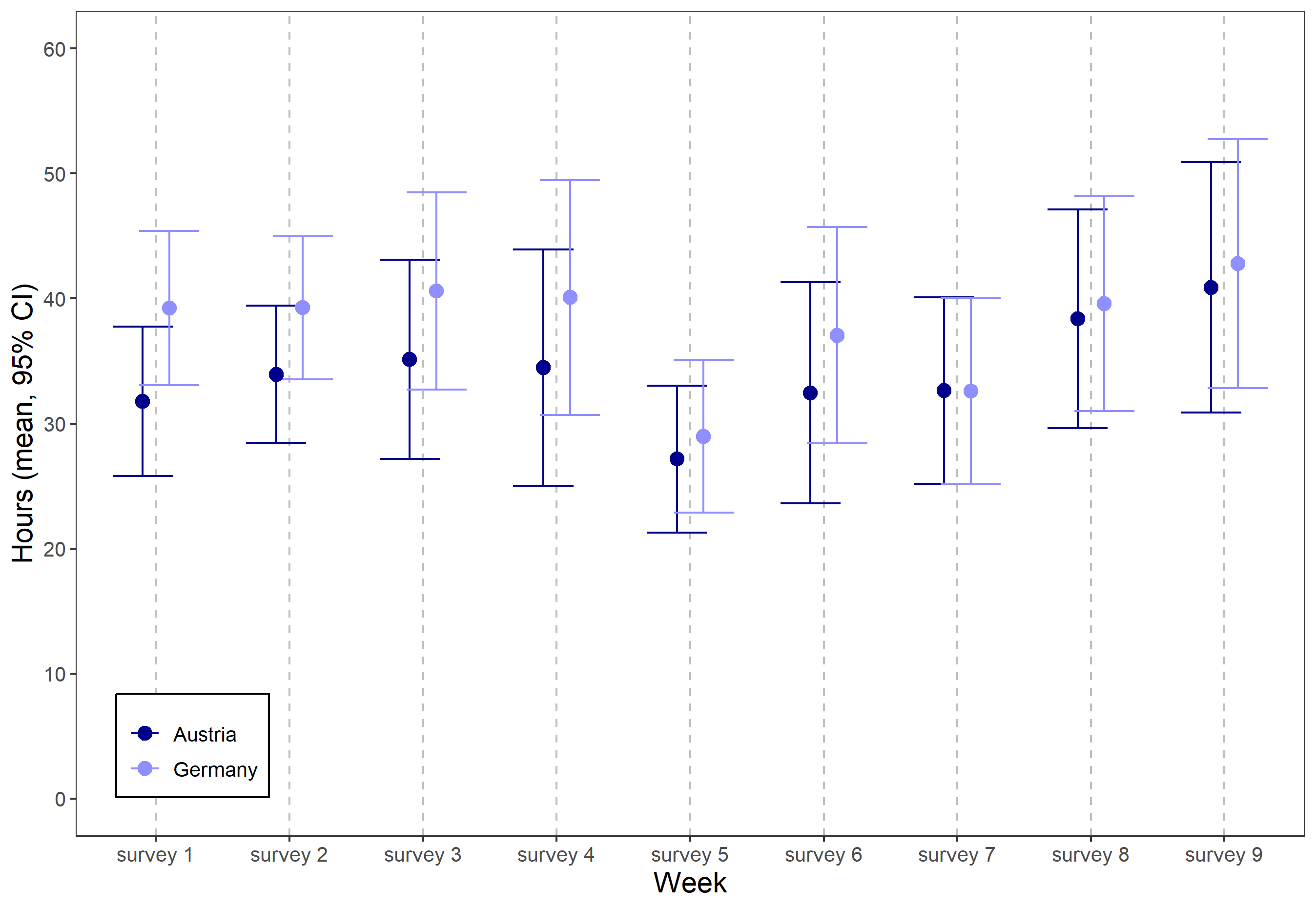  week * country | | 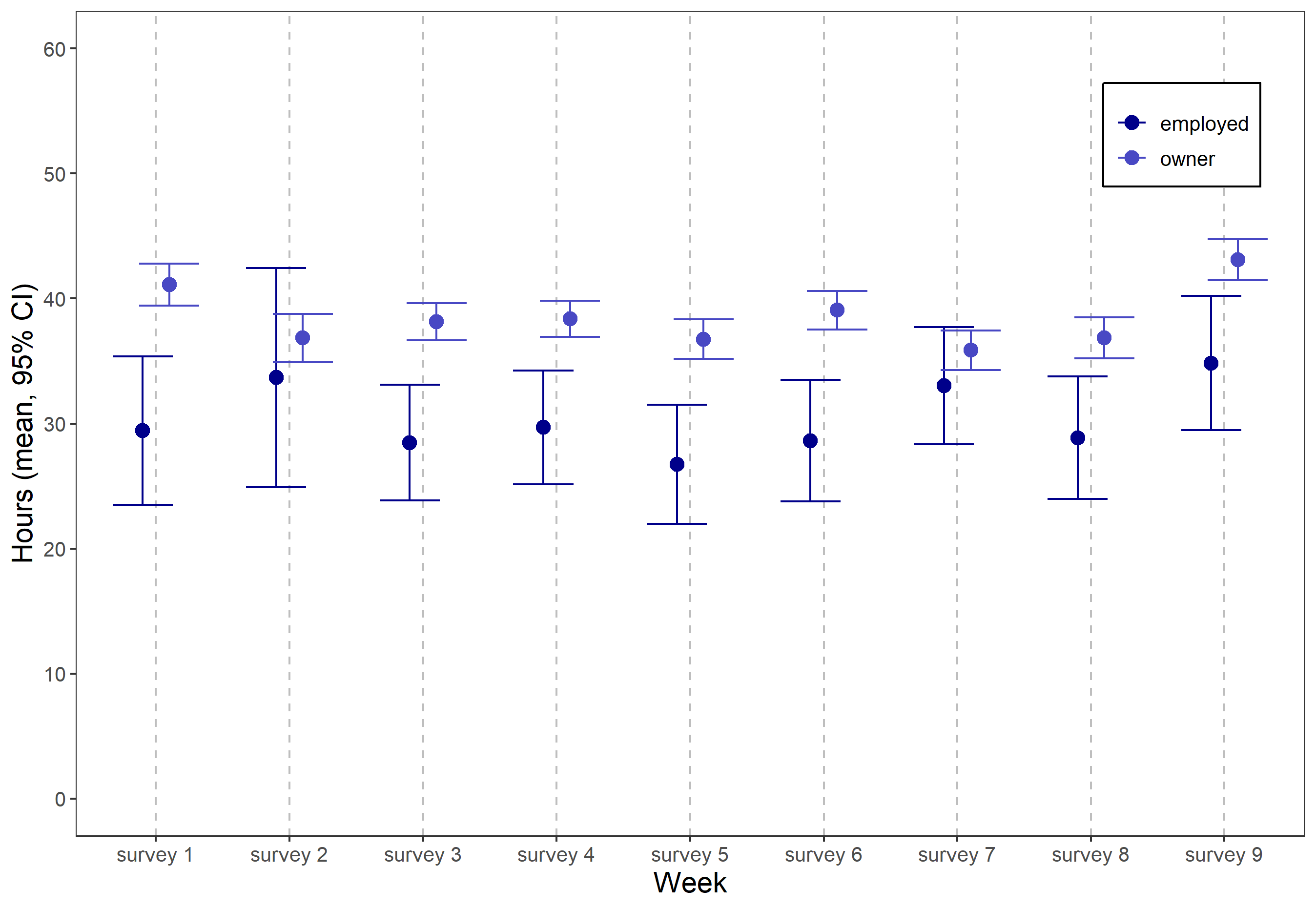  position | | n.s. |
| What proportion of your overall working time did you spend on telephone consultations? | | | | | | |
| 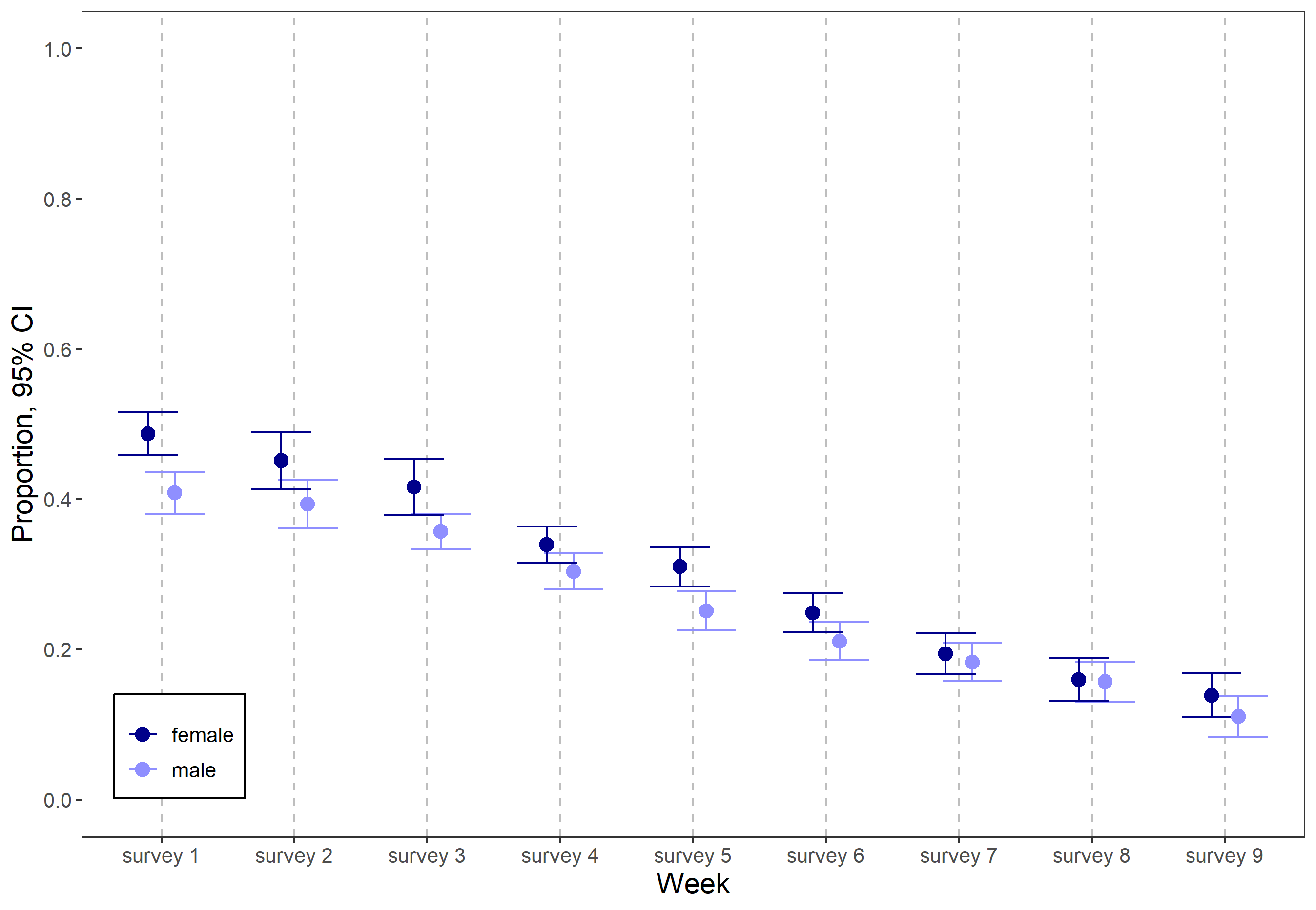  Sex | | 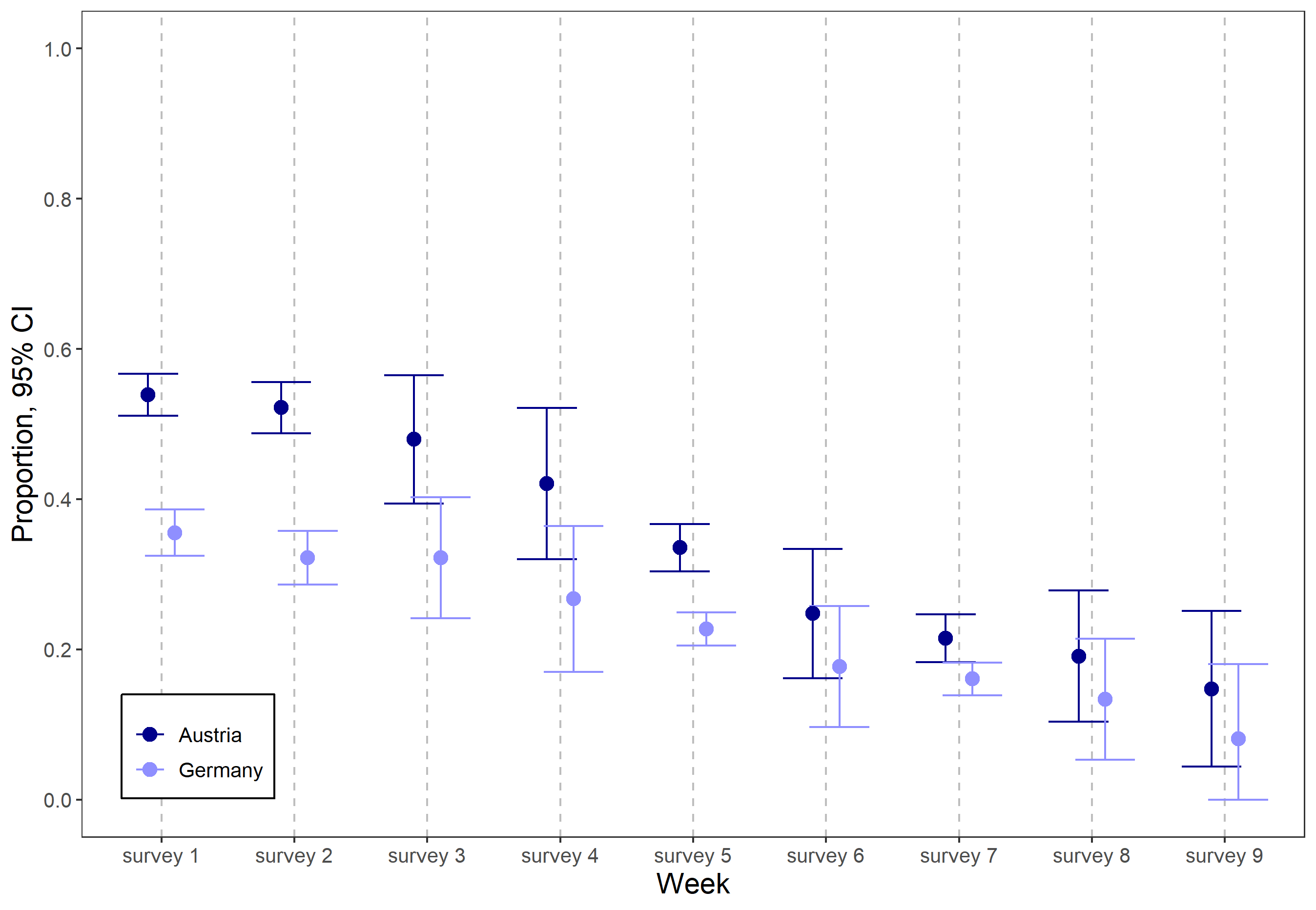  Country  week * country | | n.s. | | n.s. |
| What proportion of your overall working time did you spend on practice consultations? | | | | | | |
| n.s. | 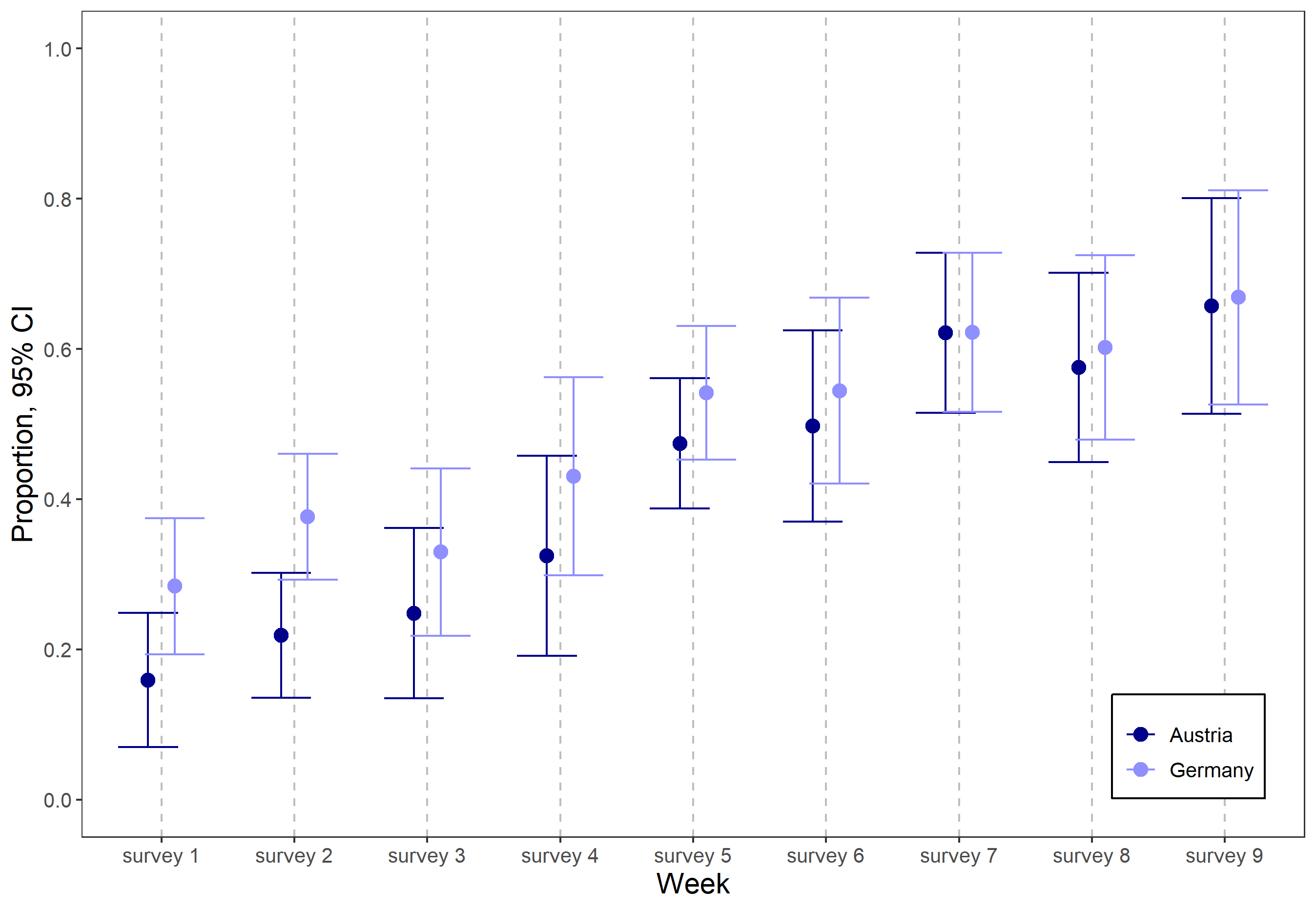  Country  week * country | | n.s. | | n.s. | |
| Sex  week*sex | Country  week * country | | position  week*position | | size of town  week*size of town | |
| What proportion of your overall working time did you spend on coordination and organization? | | | | | | |
| n.s. | 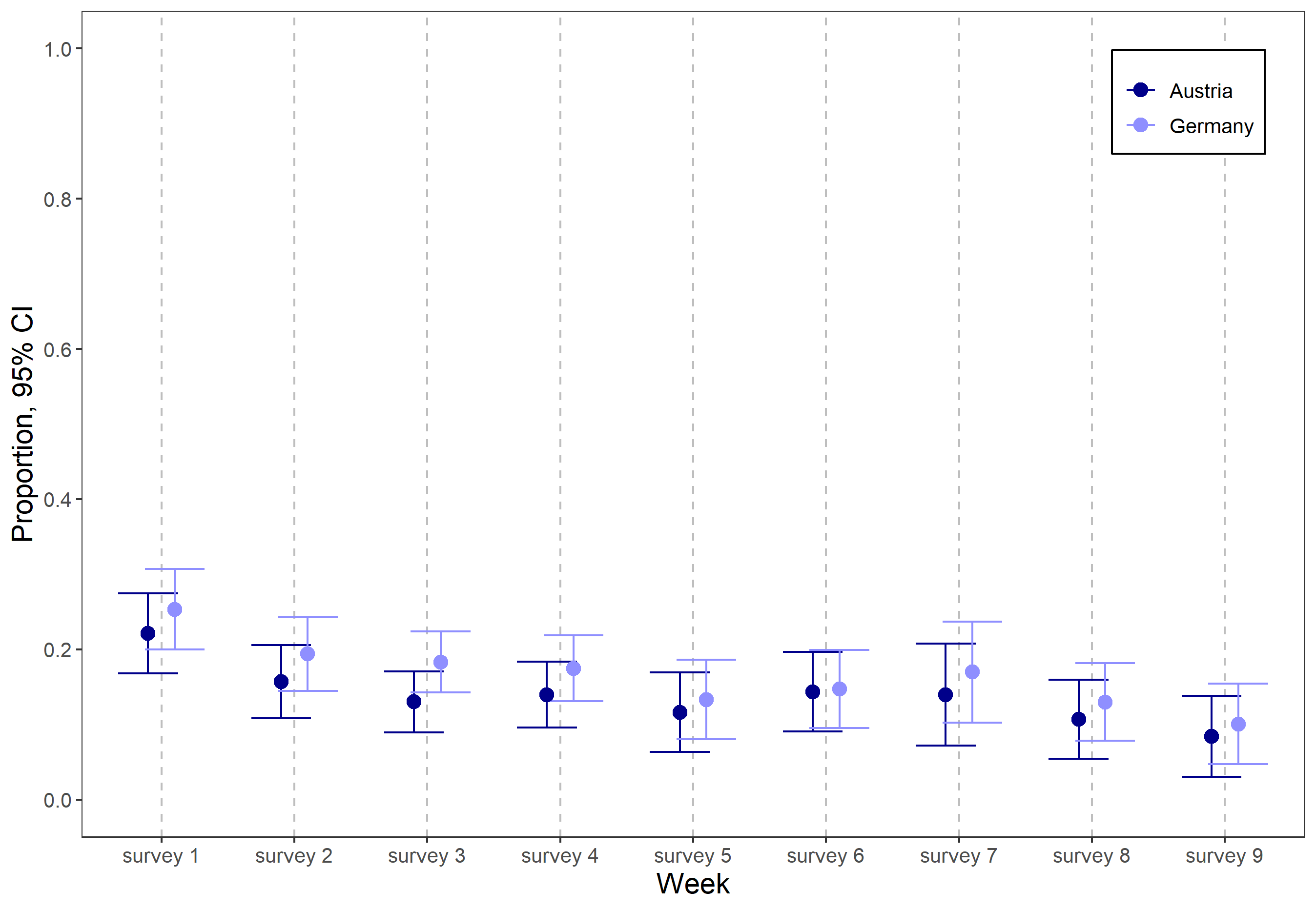  Country | | 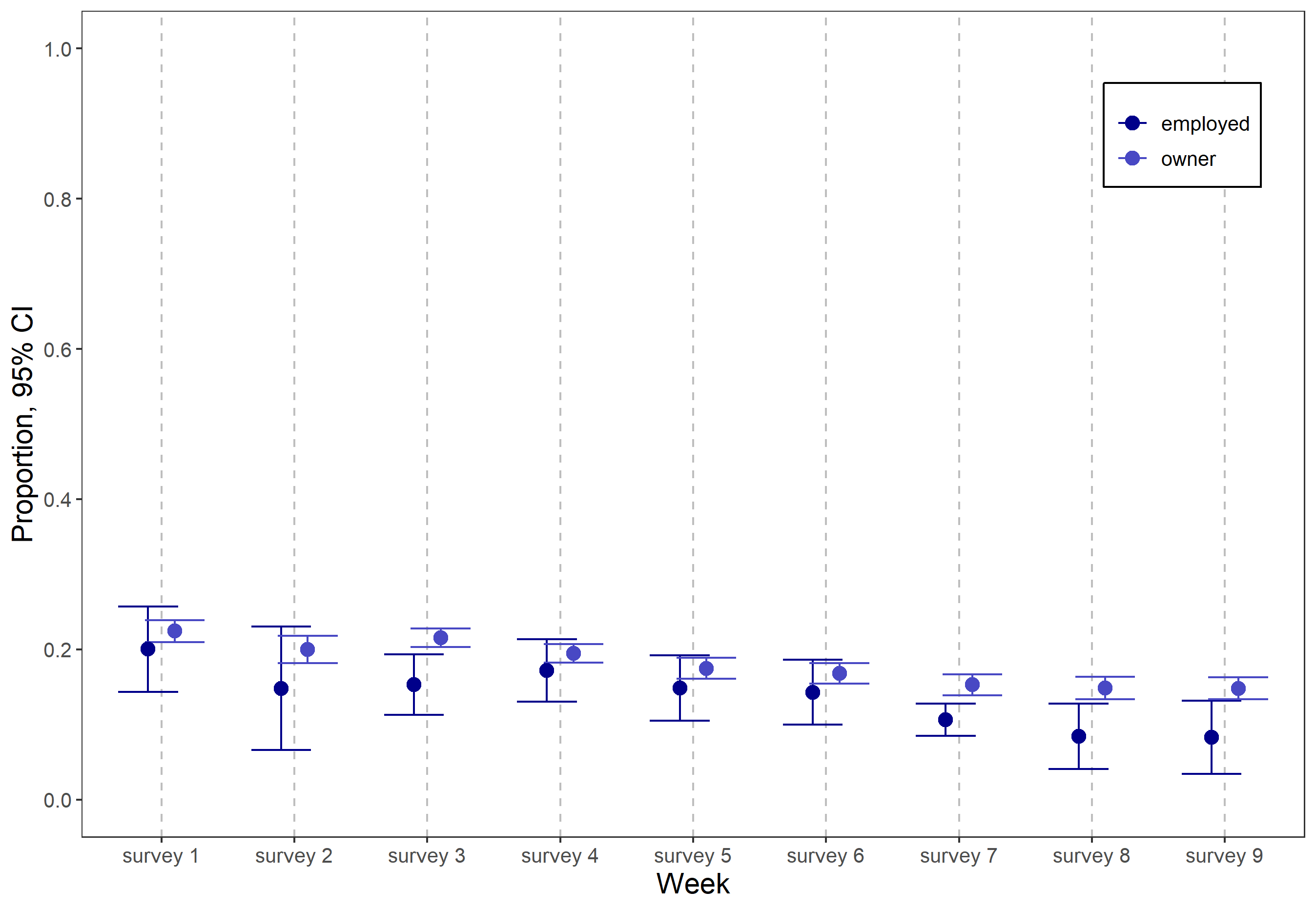  position | | 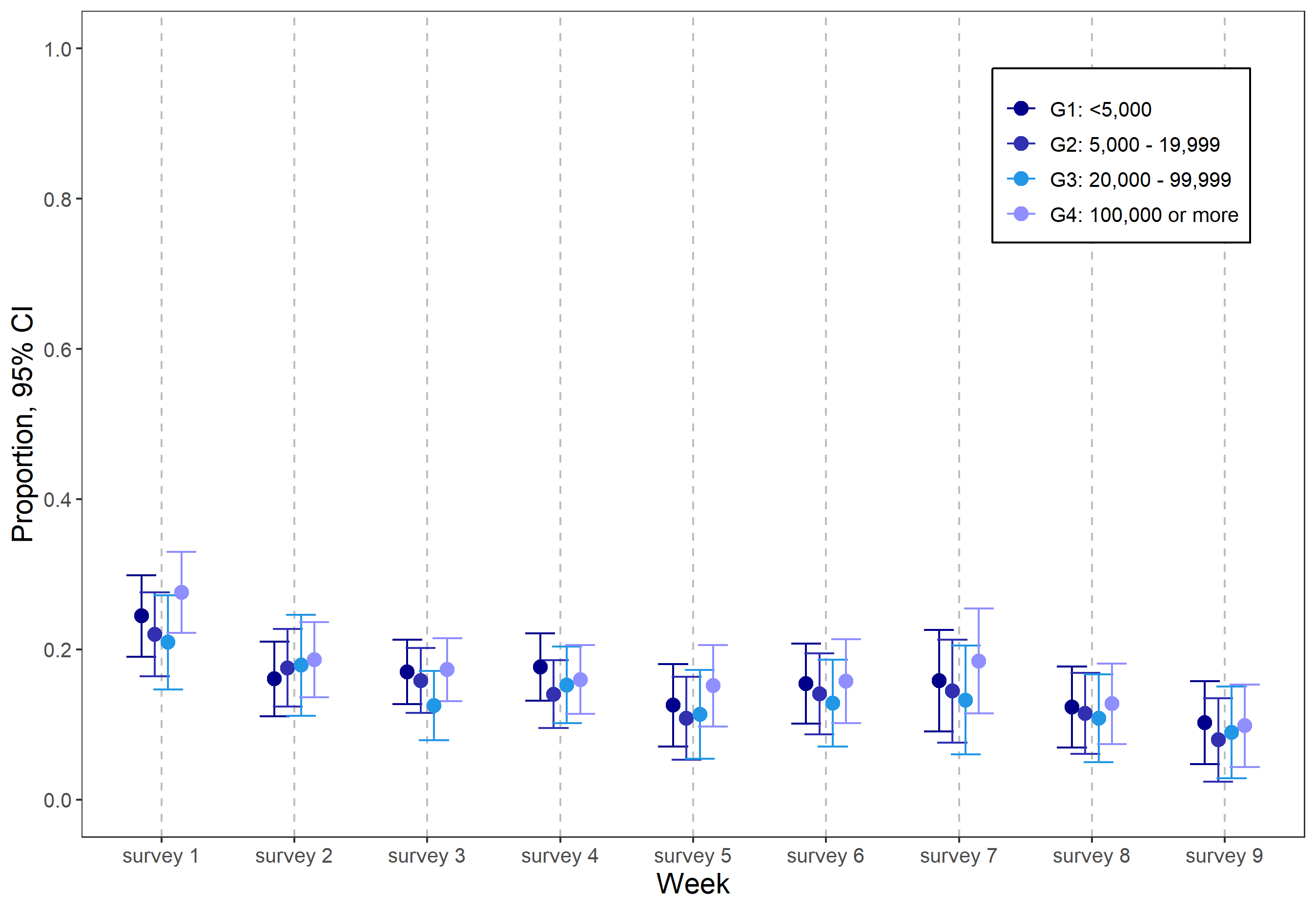  size of town | |
| How much of your overall working time was directly or indirectly linked to Covid-19? | | | | | | |
| 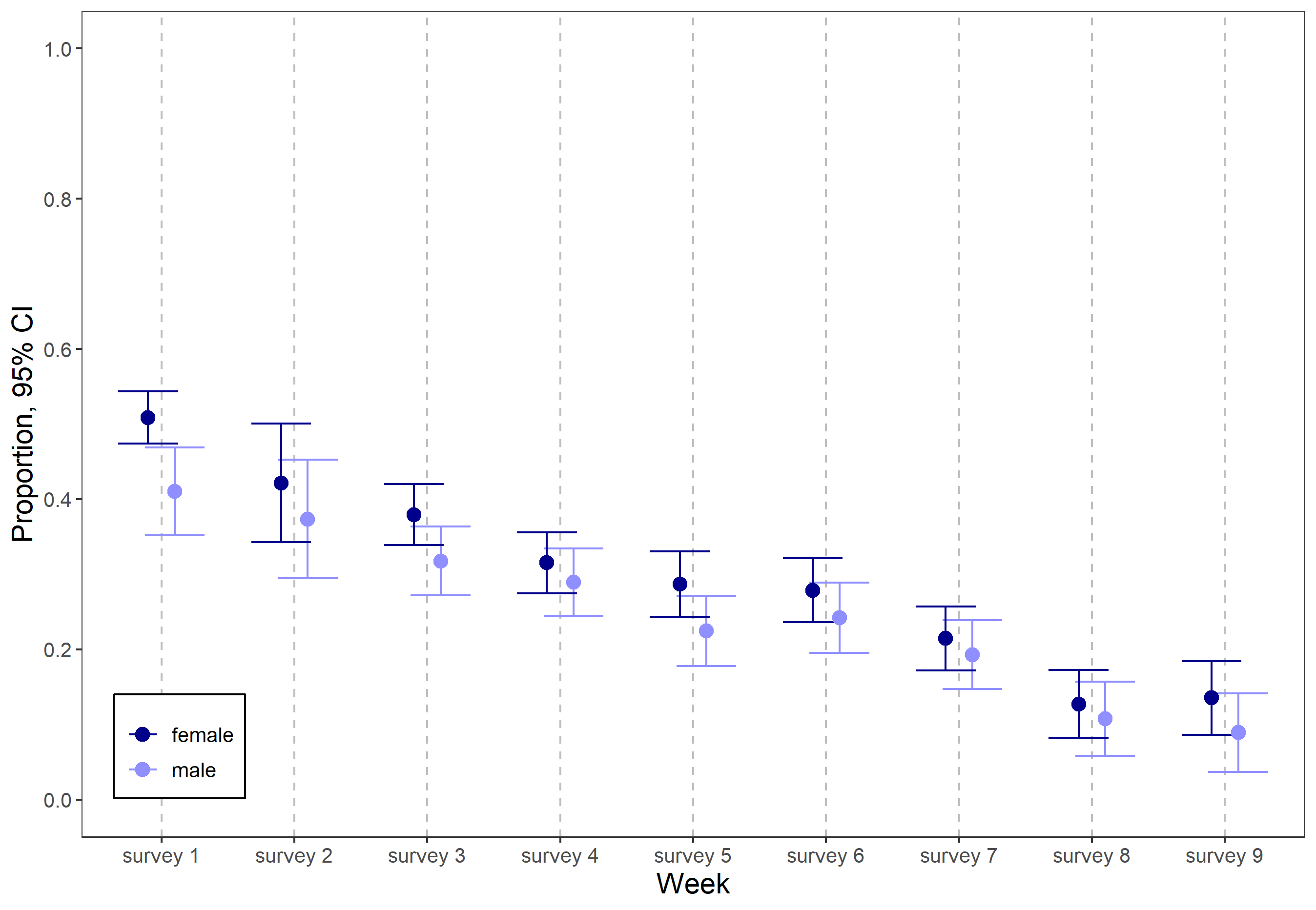 Sex | 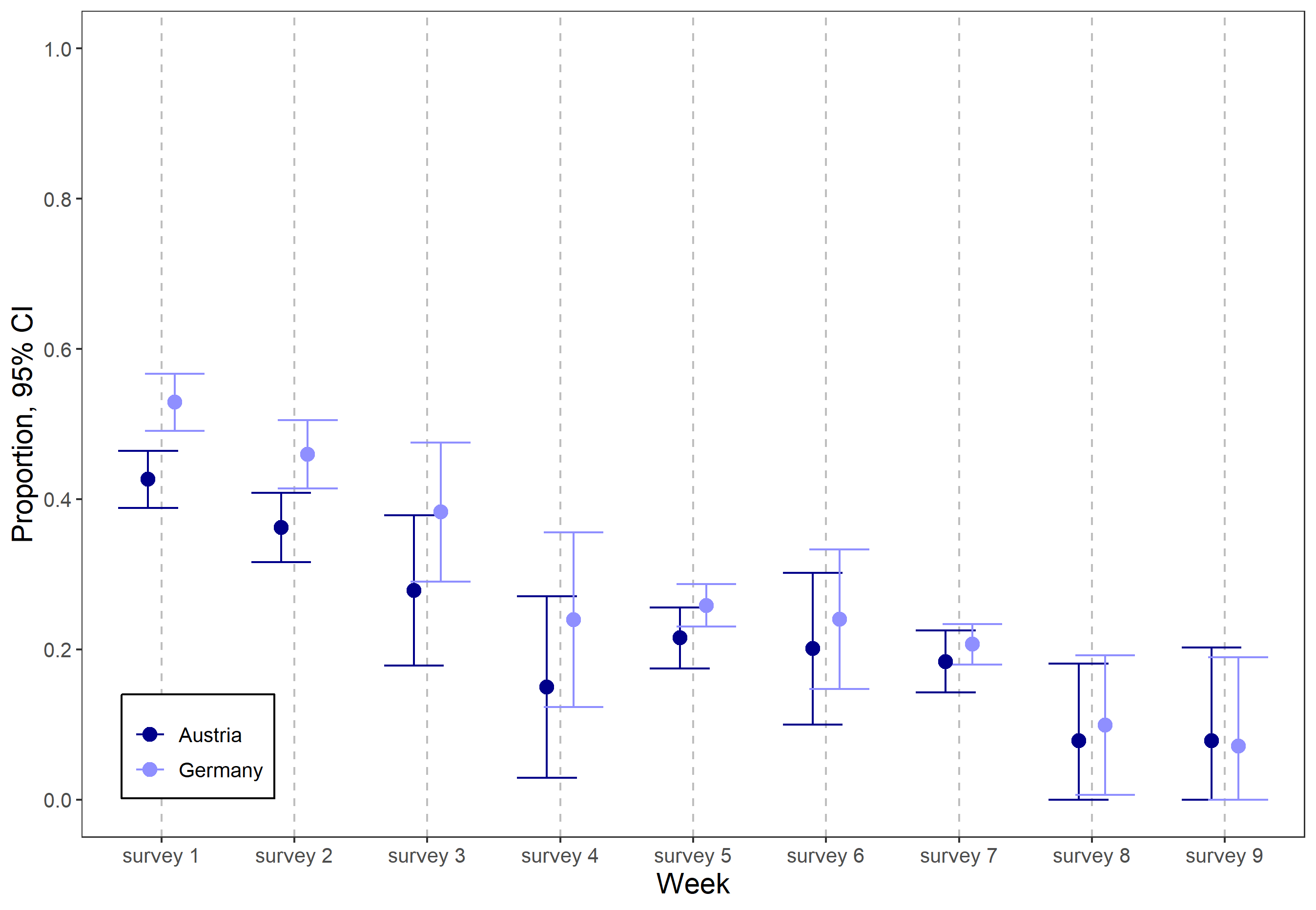 Country | | n.s. | | 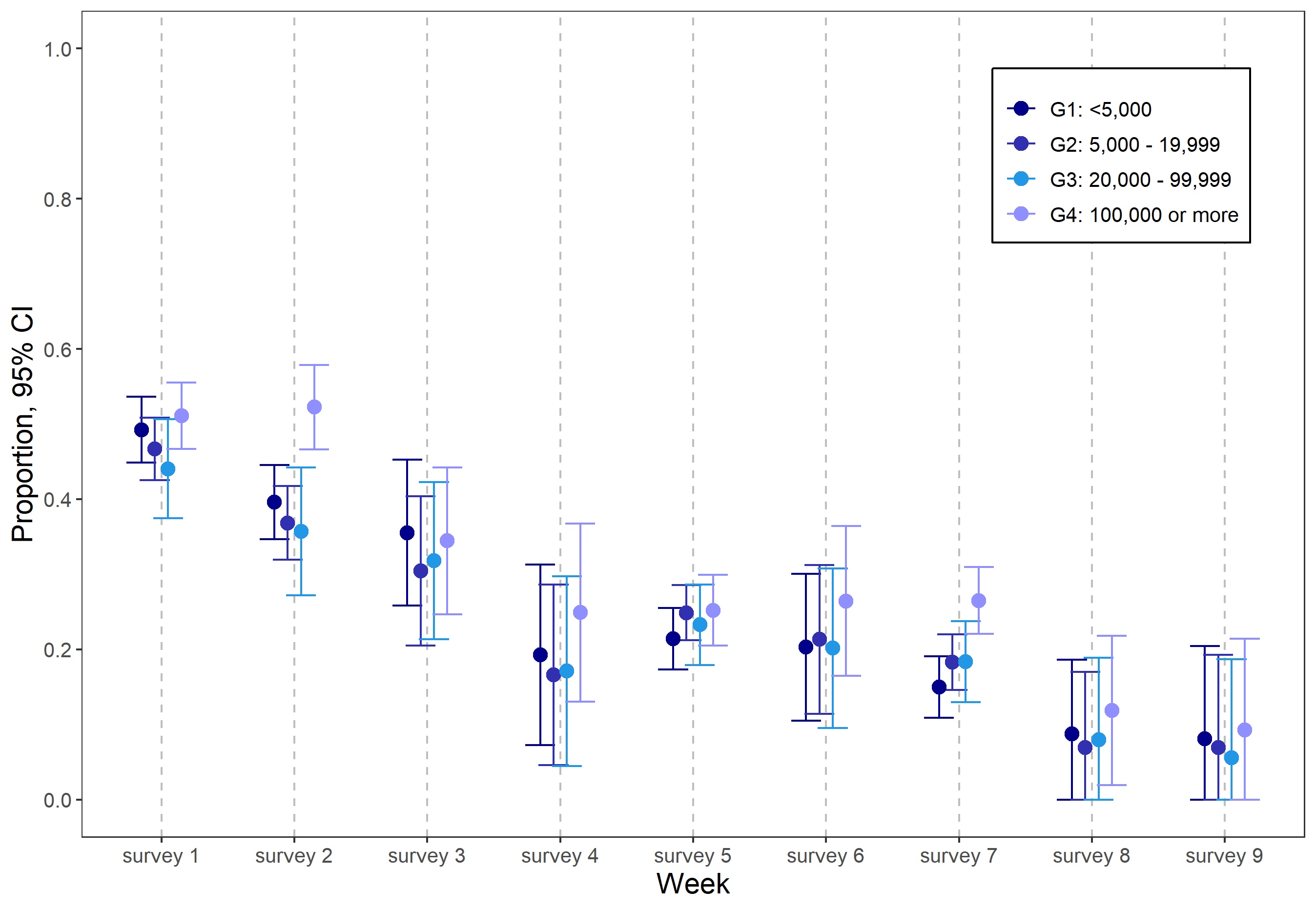 size of town | |
| How much of your overall working time was spent on routine care such as screening or treating chronically ill patients? | | | | | | |
| 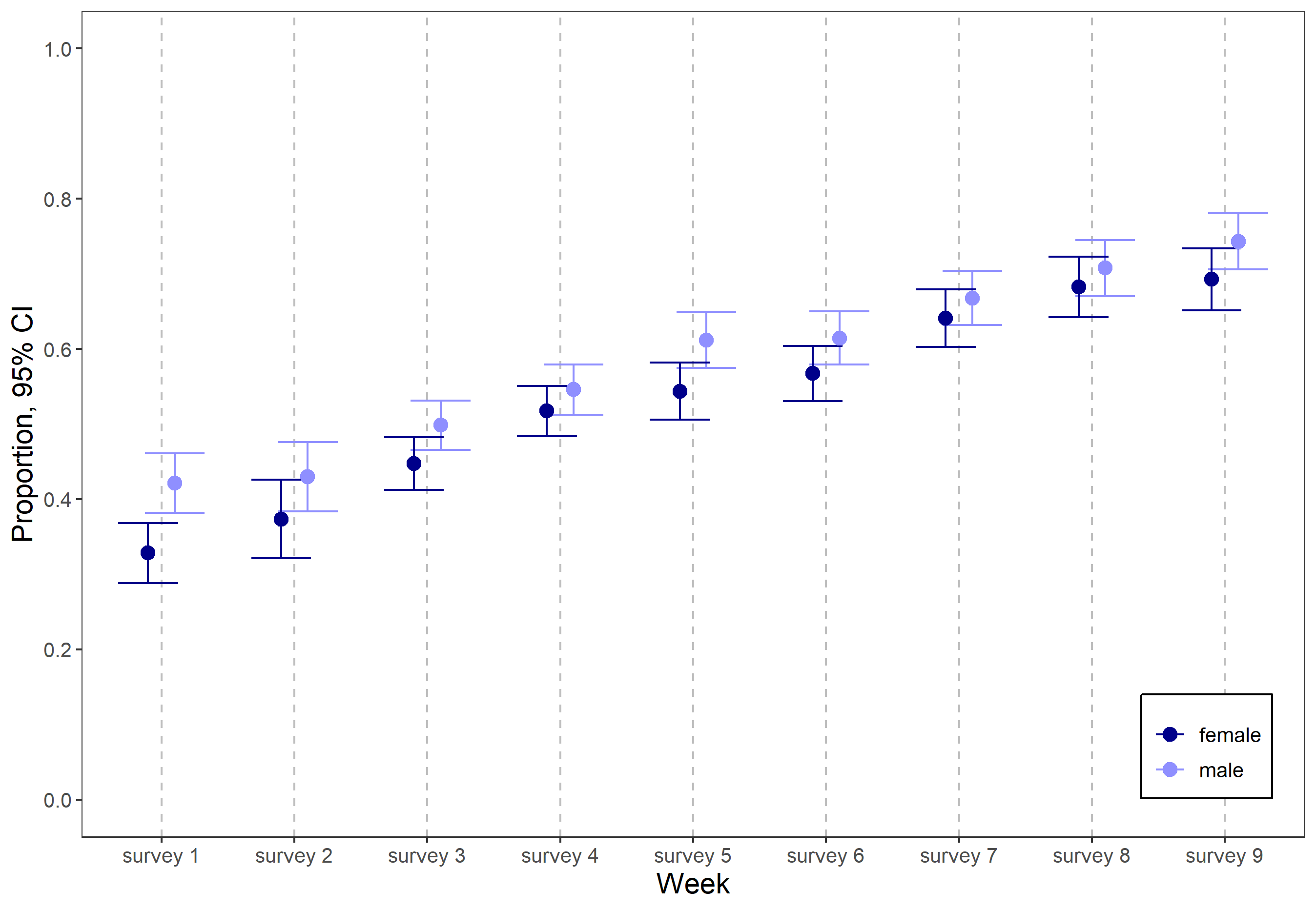  Sex | 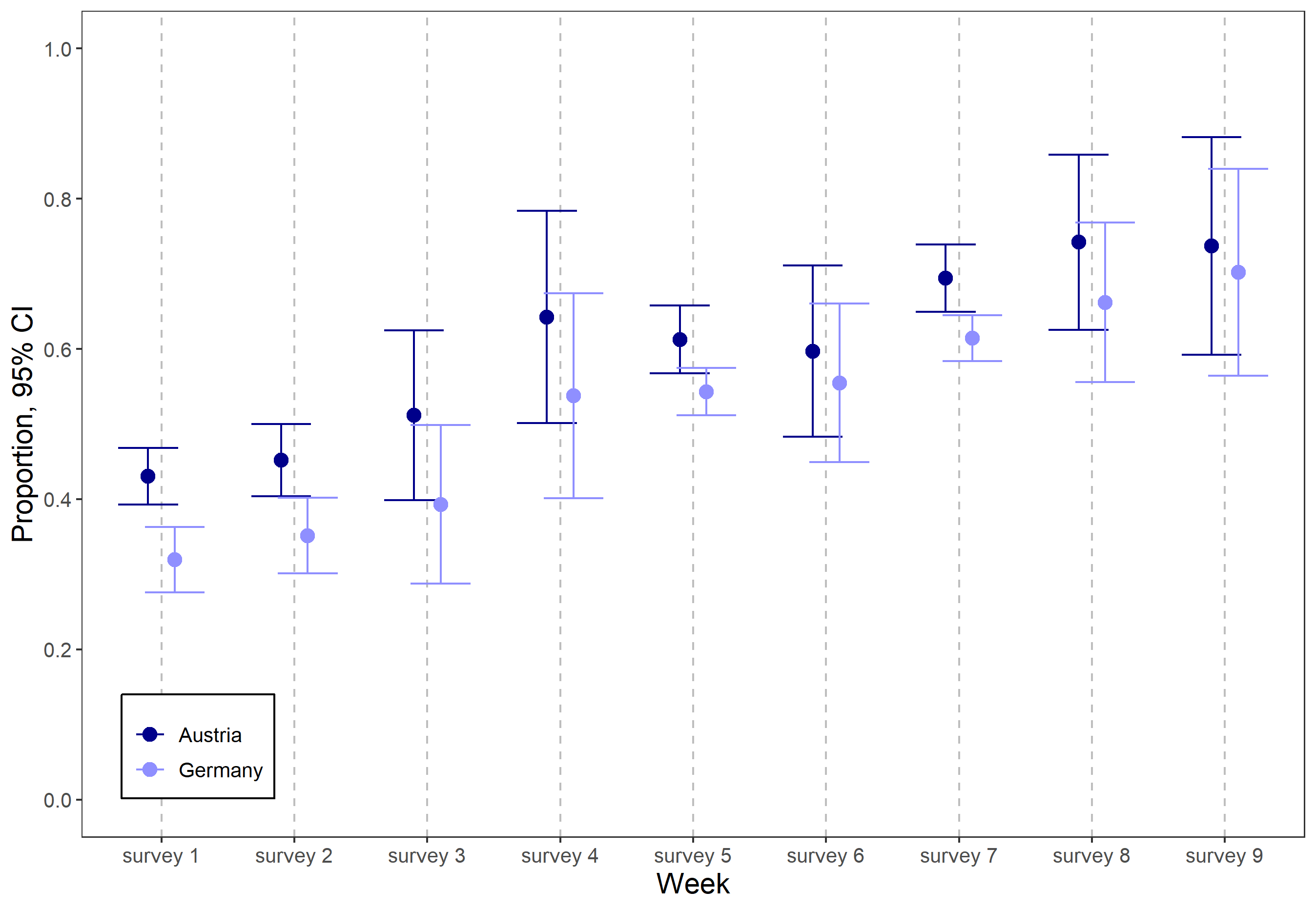  Country | | n.s. | | n.s. | |

Supplemental Figure 2.

Courses of GPs-patient and colleagues interaction over time. Significant differences in the main effects sex, country, position and size of town and significant different courses depending on these variables (interaction) are shown. It is indicated below each figure whether the main effect or the interaction is significant. If no significance was observed, no figure is shown.

| Sex  week*sex | Country  week * country | position  position*country | size of town  week*size of town |
| --- | --- | --- | --- |
| I contact patients that are quarantined at home in order to monitor the progression of the disease | | | |
| . n.s. | 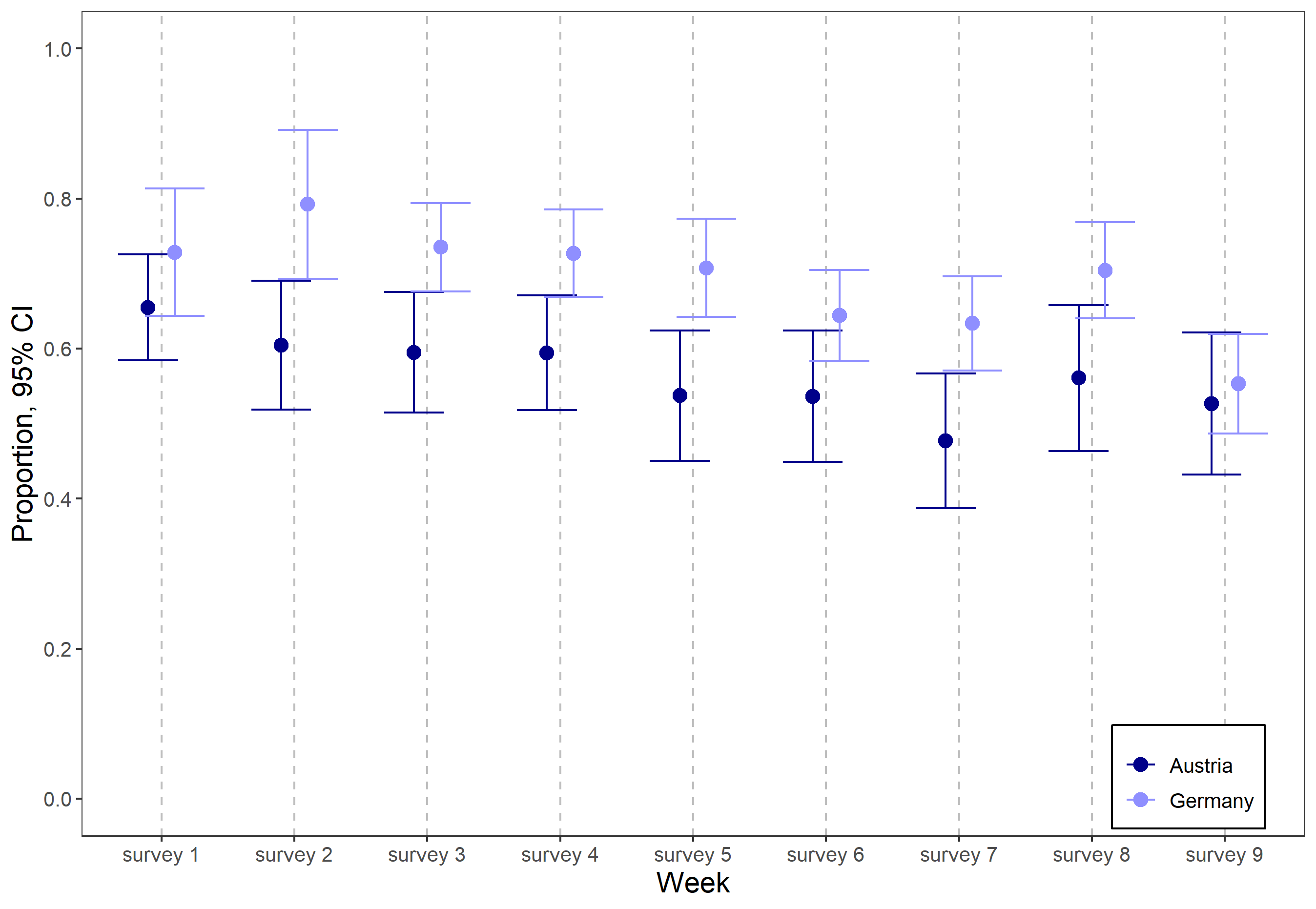  Country | n.s. | n.s. |
| I have to look after more patients because other health care services (specialists, hospitals) are less available. | | | |
| n.s. | 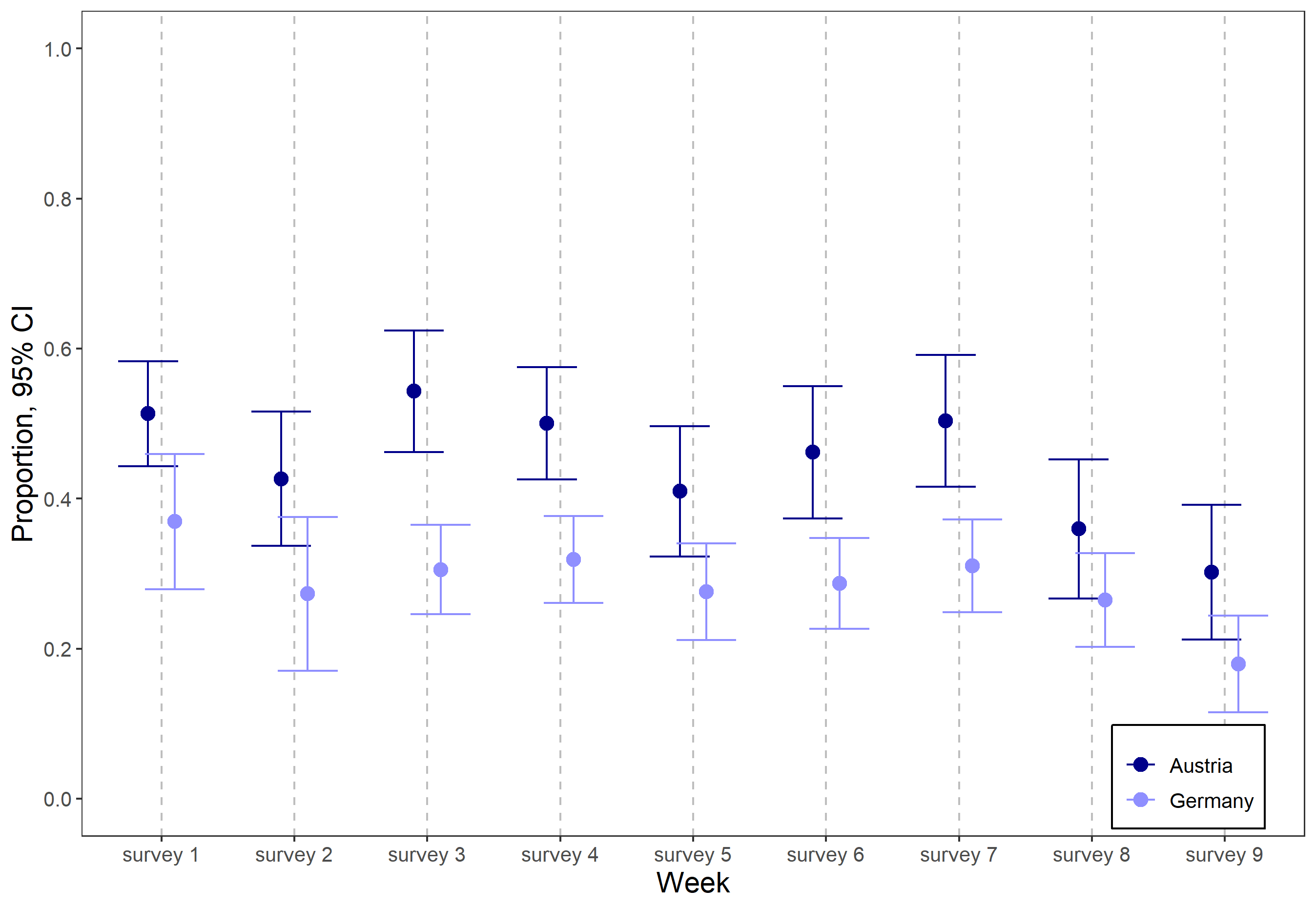  Country | n.s. | n.s. |
| I am currently treating patients that I would normally refer to specialists or to hospital. | | | |
| n.s. | 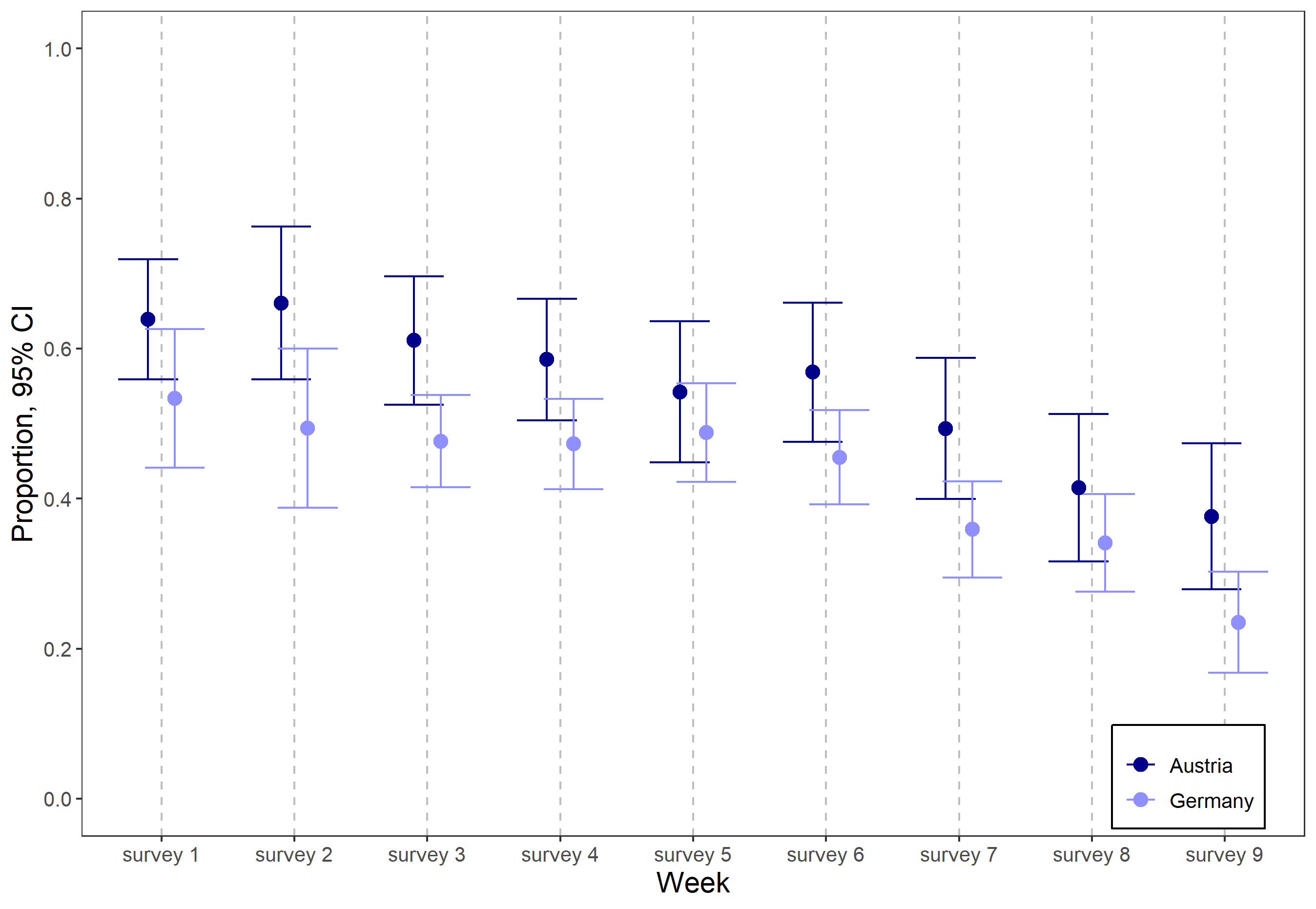  Country | n.s. | n.s. |

Supplemental Figure 2.

Courses of GP’s self-confidence and worries over time. Significant differences in the main effects sex, country, position and size of town and significant different courses depending on these variables (interaction) are shown. It is indicated below each figure whether the main effect or the interaction is significant. If no significance was observed, no figure is shown.

| Sex  week*sex | Country  week * country | position  position*country | size of town  week*size of town |
| --- | --- | --- | --- |
| I am worried that I may unknowingly infect my patients. | | | |
| n.s. | 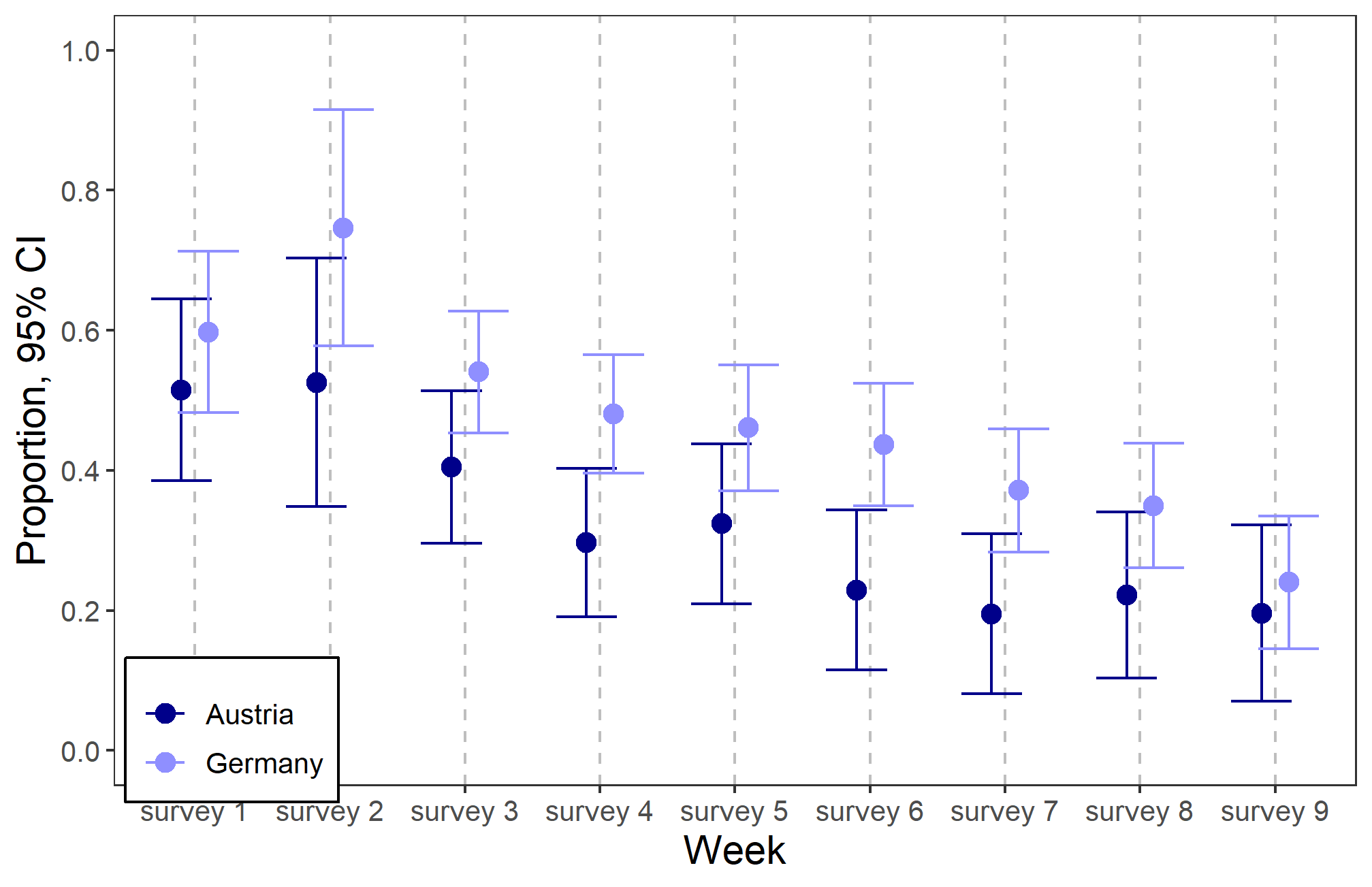  Country | n.s. | n.s. |
| When looking after patients that have been infected with Covid-19, I am sometimes unsure that I am doing everything right. | | | |
| 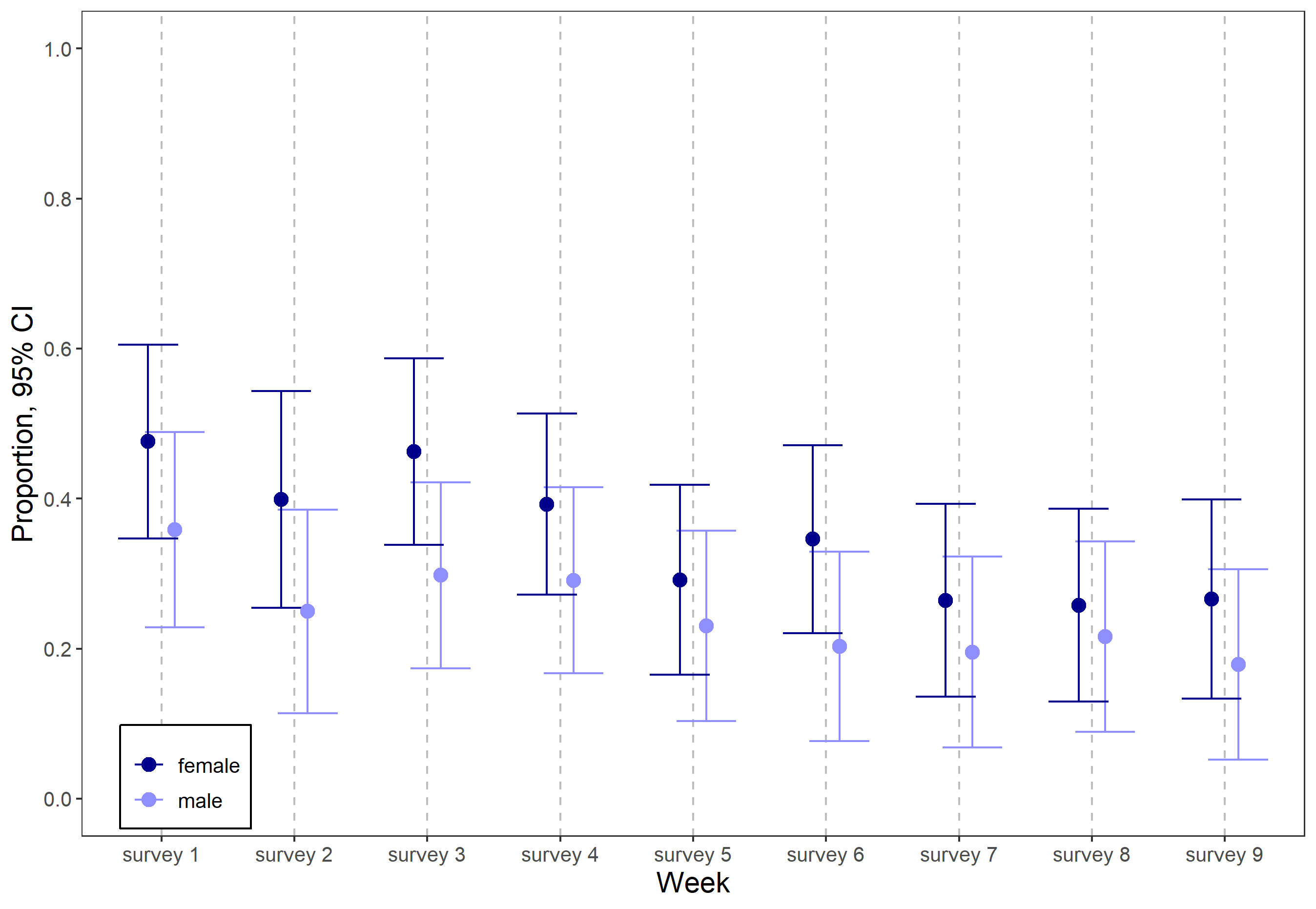  Sex | n.s. | n.s. | n.s. |
| I am worried about how the pandemic will affect the economic outlook of my employees and myself. | | | |
| 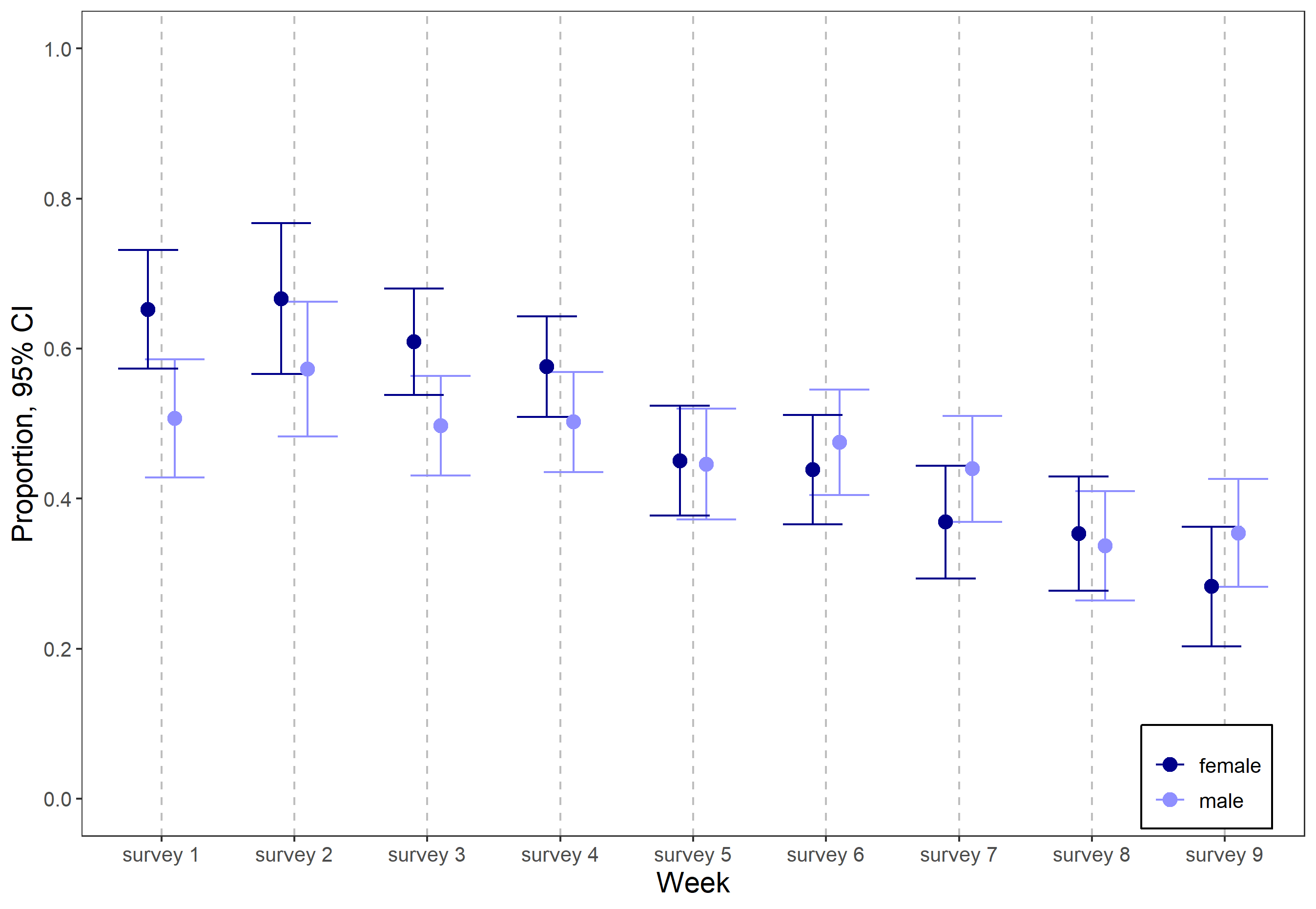  week*sex | 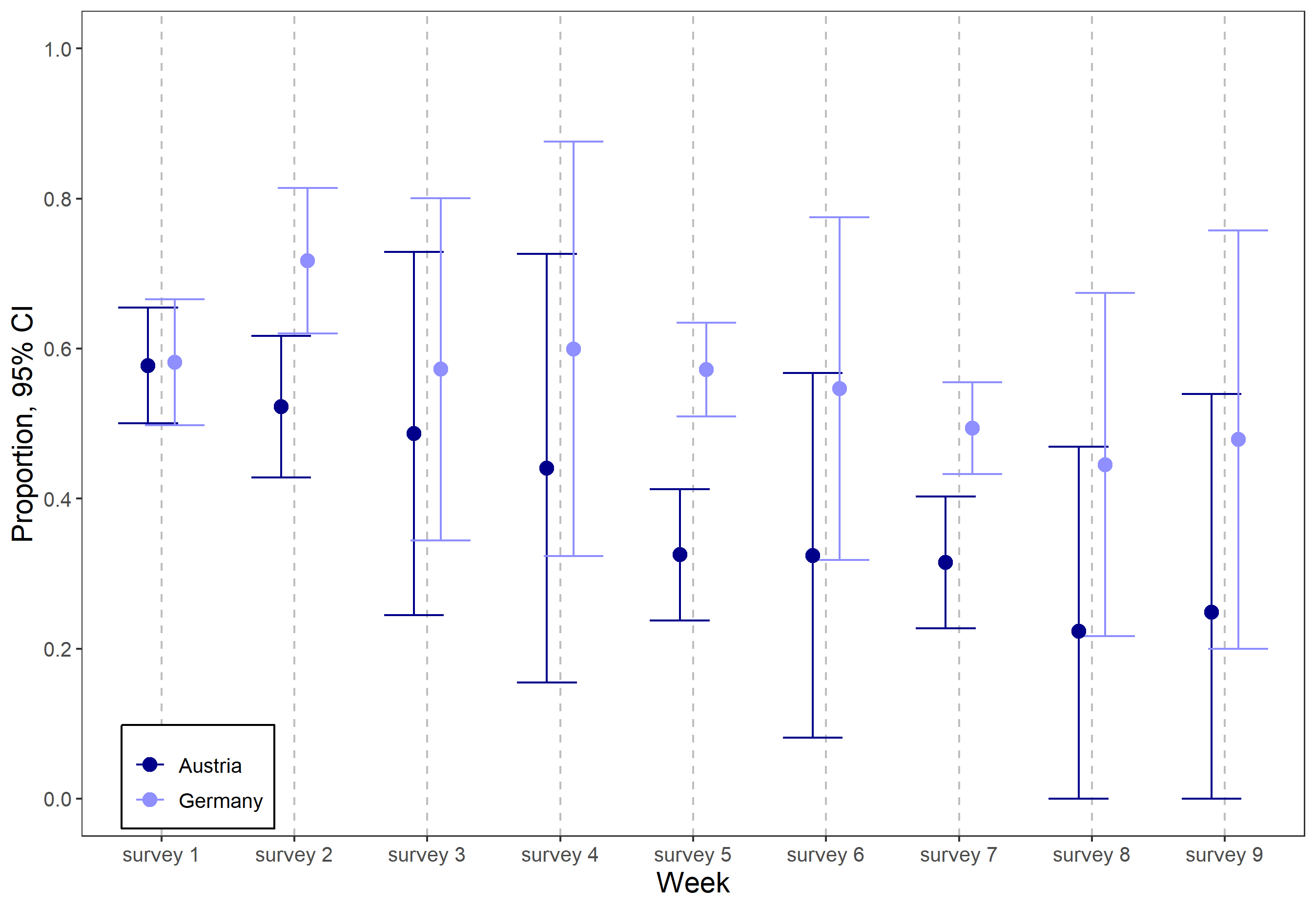  Country | n.s. | n.s. |

Supplemental Table 1 Differences between GPs answering only the baseline survey and GPs answering at least two surveys.

| Table 1.1 | p-value | GPs only answered once | GPs answering at least twice |
| --- | --- | --- | --- |
| How many hours did you work last week? | .732 | 40 (30 – 50) | 40 (30 – 50) |

| Table 1.2 | p-value | Proportion of working hours in GPs only answered once | Proportion of working hours in GPs answering at least twice |
| --- | --- | --- | --- |
| What proportion of your overall working time did you spend on telephone consultations? | .807 | 35 (20 – 60) | 30 (20 – 55) |
| What proportion of your overall working time did you spend on practice consultations? | <.001 | 30 (20-50) | 25 (10 – 45) |
| What proportion of your overall working time did you spend on coordination and organization? | <.001 | 20 (10-25) | 20 (15 – 30) |
| How much of your overall working time was directly or indirectly linked to Covid-19? | .001 | 30 (20 – 50) | 40 (20 – 60) |
| How much of your overall working time was spent on routine care such as screening or treating chronically ill patients? | <.001 | 47 (30 – 60) | 40 (20 – 60) |

| Table 1.3 | p-value | agreement in GPs only answered once | agreement in GPs answering at least twice |
| --- | --- | --- | --- |
| I contact patients that are quarantined at home in order to monitor the progression of the disease. | .037 | 67.6% | 72.4% |
| I have to look after more patients because other health care services (specialists, hospitals) are less available. | .445 | 35.0% | 36.7% |
| I am currently treating patients that I would normally refer to specialists or to hospital. | .153 | 51.4% | 54.8% |
| I am worried that I may unknowingly infect my patients. | .500 | 55.2% | 56.7% |
| When looking after patients that have been infected with Covid-19, I am sometimes unsure that I am doing everything right. | .001 | 29.1% | 36.7% |
| I am worried about how the pandemic will affect the economic outlook of my employees and myself. | .280 | 60.9% | 58.4% |
